# The Efficacy and Safety of Four Novel PCSK9 Monoclonal Antibodies in Patients With Hypercholesterolemia: A Systematic Review With Network Meta‐Analysis and Trial Sequential Analysis

**DOI:** 10.1155/cdr/6345873

**Published:** 2026-01-24

**Authors:** Sihua Wang, Chenyu Li, Duncong Fan

**Affiliations:** ^1^ Department of Pharmacy, Haining People′s Hospital (The Affiliated Haining Hospital of Jiaxing University), Haining, China; ^2^ Department of Pharmacy, Haining Hospital of Traditional Chinese Medicine, Haining, Zhejiang, China

**Keywords:** ebronucimab, hypercholesterolemia, network meta-analysis, ongericimab, recaticimab, tafolecimab

## Abstract

**Aim:**

This network meta‐analysis (NMA) evaluated four novel proprotein convertase subtilisin/kexin type 9 (PCSK9) monoclonal antibodies for hypercholesterolemia management, comparing their lipid‐lowering efficacy and safety.

**Methods:**

We systematically identified randomized controlled trials employing the frequentist NMA method to assess reductions in low‐density lipoprotein cholesterol (LDL‐C), apolipoprotein B (ApoB), and lipoprotein (a) (Lp[a]), alongside treatment‐emergent adverse events (TEAEs) and serious TEAEs. *P*‐scores ranked therapeutic hierarchies, with meta‐regression and subgroup analyses exploring heterogeneity. Trial sequential analysis determined the adequacy of cumulative evidence. Confidence in the network meta‐analysis approach was used to evaluate the confidence in the findings from NMA.

**Results:**

A total of eight trials with 3,975 Chinese patients were included. Ongericimab 150 mg every 2 weeks (Q2W) ranked first in all efficacy outcomes, demonstrating pronounced effects in LDL‐C, ApoB, and Lp(a) reduction versus placebo, with mean differences of −74.21% (95% confidence interval [CI]: −79.69% to −68.73%), −64.36% (95% CI: −68.58% to −60.13%), and −50.93% (95% CI: −56.24% to −45.61%), respectively. All interventions exhibited safety profiles comparable with placebo, with no significant differences in TEAEs or serious TEAEs incidence. The analyses suggested that a portion of the evidence base was robust and reliable.

**Conclusion:**

These findings positioned ongericimab 150 mg Q2W as a clinically optimal PCSK9 inhibitor with robust lipid‐lowering capacity. The results highlight the potential of next‐generation PCSK9 monoclonal antibodies, particularly in East Asian populations, while underscoring the need for large‐scale multinational trials to validate ethnic‐specific responses.

## 1. Introduction

Dyslipidemia remains a principal modifiable risk factor for atherosclerotic cardiovascular disease (ASCVD), with hypercholesterolemia—particularly elevated low‐density lipoprotein cholesterol (LDL‐C)—being strongly correlated with an increased risk of developing ASCVD [1]. Contemporary guidelines from the American College of Cardiology/American Heart Association (ACC/AHA) and European Society of Cardiology/European Atherosclerosis Society (ESC/EAS) unanimously prioritize LDL‐C reduction as the cornerstone of managing dyslipidemia [2, 3]. Global cardiovascular disease (CVD) burden projections reveal alarming trajectories: age‐standardized CVD prevalence is anticipated to increase by 90.0% between 2025 and 2050, culminating in 35.6 million annual deaths by mid century [4]; the incidence of CVD in China is expected to rise steadily over the next decade, whereas the mortality rate will level off after 2024 [5]. Despite a notable decline of 34.9% in global CVD mortality from 1990 to 2022, CVD continues to be a major contributor to global mortality rates and excessive healthcare expenditures [6]. The lipid profile, especially apolipoprotein B (ApoB) and lipoprotein (a) (Lp[a]), have been identified in various studies as critical lipid targets in the prevention and treatment of CVD in recent years as well [7–12].

A variety of lipid‐lowering medications are employed to manage hyperlipidemia, with statins being regarded as first‐line lipid‐modifying therapy [3], though a small portion of patients exhibit suboptimal response or intolerance, particularly at high‐intensity dosing [13]. Consequently, this therapeutic gap prompted guideline‐endorsed integration of proprotein convertase subtilisin/kexin type 9 (PCSK9) inhibitors, which enhance hepatic LDL receptor recycling via PCSK9 neutralization, achieving revolutionized lipid management [14]. Currently, alirocumab and evolocumab, two PCSK9 inhibitors, have been extensively utilized in clinical practice, with their effectiveness documented in numerous studies [15].

The therapeutic landscape evolved markedly with China′s National Medical Products Administration approving four novel PCSK9 monoclonal antibodies: tafolecimab (August 2023), ebronucimab (September 2024), ongericimab (October 2024), and recaticimab (January 2025). However, prior systematic reviews and meta‐analyses had predominantly concentrated on alirocumab and evolocumab [16, 17]. Due to the absence of head‐to‐head trials between these four agents, this study is aimed at comprehensively assessing the efficacy and safety of four novel PCSK9 monoclonal antibodies in the treatment of hypercholesterolemia employing network meta‐analysis (NMA) by integrating direct and indirect evidence in dosage levels.

## 2. Methods

### 2.1. Protocol and Registration

This systematic review was conducted in accordance with the Preferred Reporting Items for Systematic Reviews and Meta‐Analyses (PRISMA) guidelines [18] (Table S1) and following a protocol established a priori (PROSPERO: CRD42025646055). This article was based on previously conducted studies and did not contain any new studies with human participants or animals performed by any of the authors.

### 2.2. Eligibility Criteria

PICOS study selection criteria were applied for this systematic review. The full selection criteria are presented in Table S2.

The phase 3 randomized controlled trials (RCTs) evaluating the efficacy and safety of treatments for hypercholesterolemia were considered as potentially eligible papers. The predetermined study eligibility criteria were [1] patients with hypercholesterolemia; [2] tafolecimab, ebronucimab, ongericimab, or recaticimab as an intervention; [3] placebo as a control group; [4] the study reported the efficacy data of the percentage change from baseline in LDL‐C, ApoB, or Lp(a), as well as safety data, including treatment‐emergent adverse events (TEAEs) or serious TEAEs; [5] the study was a phase 3 RCT. The exclusion criteria were as follows: [1] studies without available data; [2] duplicated reports; [3] conference articles or abstracts.

### 2.3. Search Strategy

A systematic search was conducted for relevant articles in Cochrane Library, PubMed, Embase, Web of Science, and ClinicalTrials.gov to 1 February 2025, with no language restrictions applied, in order to ensure identification of all potentially relevant studies. The search strategy was adapted for each database and register. The search items included medical subject headings and the keywords such as “tafolecimab,” “randomized controlled trial,” and “hypercholesterolemia.” The full strategy is shown in Table S3.

### 2.4. Study Selection and Data Extraction

We reviewed all potentially eligible papers against eligibility criteria. The Zotero software was employed for the purpose of importing the retrieved references and piloting of the study selection process. Two reviewers dual screened the abstracts with conflict resolution; another reviewer screened all excluded abstracts and, if needed, resolved conflicts. In addition, one reviewer screened all included full‐text articles and a second reviewer screened all excluded full‐text articles. Extracts from eligible RCTs for review included [1] trial name and year, [2] number of patients, [3] characteristics of patients (age, gender, and lipid profiles at baseline), [4] treatment regimens, [5] duration of follow‐up, and [6] efficacy and safety data of interest. One reviewer extracted data using a piloted form and another reviewer checked for correctness and completeness of extracted data. Any discrepancies were resolved by a third investigator for the final decision.

### 2.5. Risk of Bias Assessment

The qualities of the eligible studies were assessed using the Cochrane risk of bias 2 instrument [19]. The main assessment content included [1] bias arising from the randomization process, [2] bias due to deviations from the intended interventions, [3] bias due to missing outcome data, [4] bias in measurement of the outcome, [5] bias in selection of the reported result. A series of signaling questions were employed in each domain to elicit information pertinent to an assessment of risk of bias. The risk‐of‐bias judgments for each domain were categorized as “low risk of bias,” “some concerns,” or “high risk of bias.” These judgments were derived from and summarized by the responses to the signaling questions. The response options for an overall risk‐of‐bias judgment were consistent with those for individual domains. The overall risk of bias was typically commensurate with the worst risk of bias in any of the domains; however, if a study was judged to have “some concerns” about risk of bias for multiple domains, it might be judged as at high risk of bias overall.

### 2.6. Synthesis

A frequentist NMA was performed using the “netmeta” package (R Version 4.4.1) to compare the efficacy and safety of dosage levels across four agents for hypercholesterolemia [20]. Continuous outcomes were expressed as mean differences (MDs) with 95% confidence intervals (CIs), whereas dichotomous outcomes were reported as risk ratios (RRs) with 95% CIs. The assumption of coherence across studies with differing treatment sets was evaluated via the design‐by‐treatment interaction model (global test). Local inconsistency between direct and indirect estimates within each closed evidence loop was examined using the node‐splitting method [21]. The random‐effects model accounted for both within‐study and between‐study variance. Heterogeneity was assessed using restricted maximum likelihood estimation to compute *τ* ^2^ and *I* ^2^ statistics [22]; consistent with Cochrane Handbook guidelines, *I* ^2^ values ≥ 50% indicated substantial heterogeneity, and values ≥ 75% denoted considerable heterogeneity [23]. Treatment rankings were derived using *P*‐scores and visualized via rank‐heat plots [24, 25]. For analysis involving > 10 trials, publication bias was evaluated through visual inspection of funnel plots and quantified using Egger′s test [26]. Sensitivity analysis assessed the robustness of evidence by excluding high‐risk‐of‐bias studies and visually comparing forest plot magnitudes. To explore substantial heterogeneity (*I* ^2^ ≥ 50*%*), meta‐regression (implemented via the “multinma” package using quasi‐Monte Carlo integration) and subgroup analyses were conducted for the following covariates: other forms of hyperlipidemias, baseline LDL‐C level (threshold: 3.36 mmol/L [130 mg/dL]), and follow‐up duration (threshold: ≥ 24 weeks) [27].

Finally, trial sequential analysis (TSA) was applied for direct comparisons to assess the evidence robustness through calculation of the required information size (RIS) by Copenhagen Trial Units TSA Software (http://www.ctu.dk/tsa) [28]. The Holm–Bonferroni method was further applied to control the family‐wise error rate; while conservative, this adjustment ensures rigorous error control and provides a more reliable basis for subsequent clinical decision‐making [29].

### 2.7. Confidence Evaluation

CINeMA (Confidence in Network Meta‐Analysis) approach was used to evaluate the confidence in the findings from NMA, which considers six domains: within‐study bias, reporting bias, indirectness, imprecision, heterogeneity, and incoherence [30]. The judgments of certainty of evidence were conducted independently by two authors, and any divergence was resolved by discussion.

## 3. Results

### 3.1. Study Selection and Characteristics

**Figure 1:**
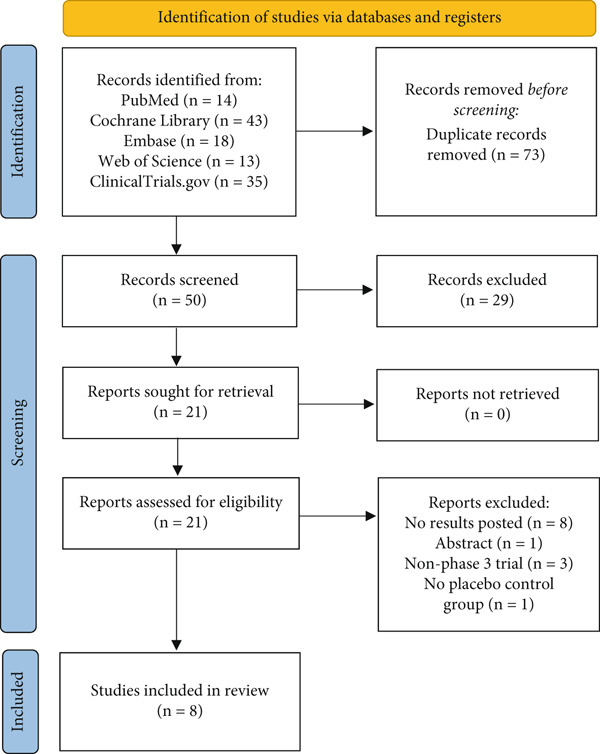
PRISMA flowchart.

We identified 123 publications through database searches, and 73 duplicated references were removed, of which 21 studies were potentially eligible after the screening of titles and abstracts, and eight studies were eligible on full‐text review for inclusion in the systematic review (Figure 1)

The characteristics of the included studies are presented in Table 1. A total of 3975 individuals with a mean age ranging from 44.45 to 59.76 years were included in these studies. Among the eligible studies, all trials reported lipid profile parameters at the end point in the Chinese population with hyperlipidemia [31–38]. Seven studies reported outcomes of TEAEs and serious TEAEs [31–36, 38]; however, one study was excluded from the NMA because it did not provide data stratified by dose groups [35]. The RCTs encompassed a total of 10 treatment regimens, including tafolecimab 150 mg every 2 weeks (Q2W), tafolecimab 450 mg every 4 weeks (Q4W), tafolecimab 600 mg every 6 weeks (Q6W), ebronucimab 150 mg Q2W, ebronucimab 450 mg Q4W, ongericimab 150 mg Q2W, ongericimab 300 mg Q4W, recaticimab 150 mg Q4W, recaticimab 300 mg every 8 weeks, and recaticimab 450 mg every 12 weeks (Q12W), in comparison with placebo. The input data on efficacy and safety for NMA are presented in Tables S4 and S5, respectively.

**Table 1:** Characteristics of eligible studies.

**Study**	**Trial registration**	**Follow-up, weeks**	**Population**	**Mean age, years**	**Female, n**	**Mean LDC-C, mmol/L**	**Interventions**
Tafolecimab versus placebo (3 RCTs)							
Huo et al. 2023 (CREDIT‐1) [31]	NCT04289285	48	Chinese patients with non‐FH on stable lipid‐lowering therapy for at least 4 weeks	57.54 (8.86)	209 (34.04)	2.85 (0.77)	TAFO 450 mg Q4W, TAFO 600 mg Q6W
Chai et al. 2023 (CREDIT‐2) [32]	NCT04179669	12 (double‐blind treatment)	Chinese patients with HeFH remain on the statins with or without ezetimibe	49.41 (12.81)	71 (47.97)	4.25 (1.11)	TAFO 150 mg Q2W, TAFO 450 mg Q4W
Qi et al. 2023 (CREDIT‐4) [33]	NCT04709536	12 (double‐blind treatment)	Chinese patients with HeFH or at high or very high cardiovascular risk with non‐FH on stable lipid‐lowering therapy	56.8 (9.2)	94 (31)	3.06 (0.9)	TAFO 450 mg Q4W
Ebronucimab versus Placebo (1 RCT)							
Zhang et al. 2024 [34]	CTR20212466 and NCT05255094	12	Chinese patients with hypercholesterolemia on a stable dose of moderate or potent statin therapy (with or without ezetimibe)	59.76 (10.09)	182 (39.48)	Not reported	EBRO 150 mg Q2W, EBRO 450 mg Q4W
Ongericimab versus Placebo (2 RCTs)							
Wang et al. 2024 [35]	NCT04781114	52	Chinese patients with primary hypercholesterolemia and mixed dyslipidemia receiving stable and optimized lipid‐lowering therapy	59.47 (9.89)	314 (39.15)	2.7 (0.98)	ONGE 150 mg Q2W, ONGE 300 mg Q4W
Zhao et al. 2024 [36]	CTR20220027	12	Chinese patients with primary hypercholesterolemia and mixed dyslipidemia on stable optimized lipid‐lowering therapy.	59.20 (10.11)	123 (48.24)	3.10 (0.90)	ONGE 150 mg Q2W
Recaticimab versus Placebo (2 RCTs)							
Xu et al. 2024 (REMAIN‐1) [37]	NCT04849000	12 or 16 (double‐blind treatment)	Chinese patients with non‐FH and mixed hyperlipemia	44.45 (12.87)	327 (46.51)	3.69 (0.58)	RECA 150 mg Q4W, RECA 300 mg Q8W, RECA 450 mg Q12W
Sun et al. 2024 (REMAIN‐2) [38]	NCT04885218	48	Chinese patients with non‐FH on statins therapy	55.86 (10.94)	245 (35.56)	2.82 (0.77)	RECA 150 mg Q4W, RECA 300 mg Q8W, RECA 450 mg Q12W

*Note:* Values are mean (SD), or *n* (%).

Abbreviations: EBRO, ebronucimab; HeFH, heterozygote familial hypercholesterolemia; LDL‐C, low‐density lipoprotein cholesterol; non‐FH, non‐familial hypercholesterolemia; ONGE, ongericimab; Q2, 4, 6, 8, 12 W, every 2, 4, 6, 8, 12 weeks; RCTs, randomized controlled trials; RECA, recaticimab; TAFO, tafolecimab.

### 3.2. Risk‐of‐Bias, Incoherence and Heterogeneity Assessments

The findings revealed that bias due to deviations from the intended interventions and selecting bias emerged as the most common categories associated with potential bias risk among the studies. The overall risk was considered “some concerns” bias rating (Figure 2). Most of the outcomes had no serious concerns for incoherence or heterogeneity (Table S6; Figures S1, S2, S3, S4, and S5). Less than 10 studies met the criteria, therefore, the detection of publication bias had not been implemented.

Figure 2Risk of bias for included studies: (a) risk of bias summary for included studies; (b) risk of bias graph of included studies.

**Figure:**
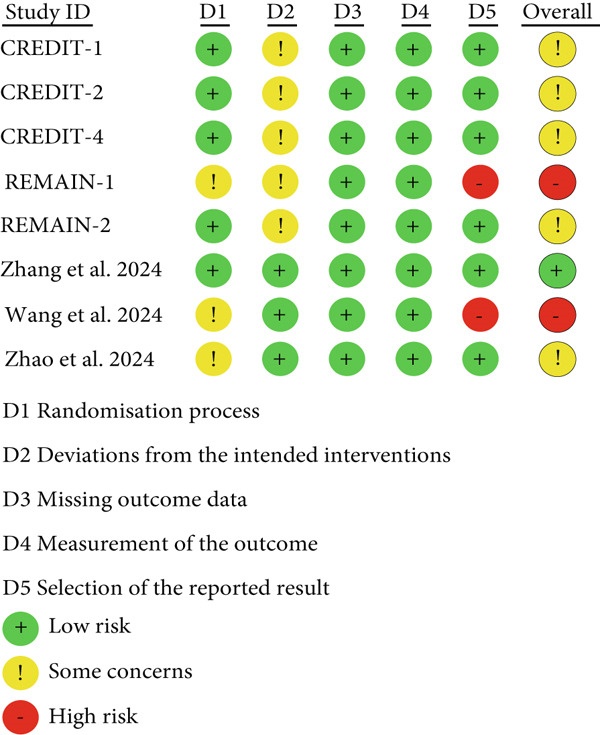
(a)

**Figure:**
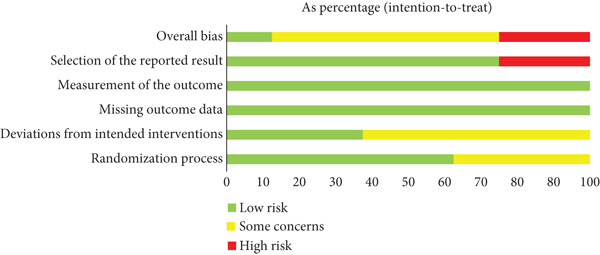
(b)

### 3.3. Network Estimates for Clinical Efficacy

**Figure 4:**
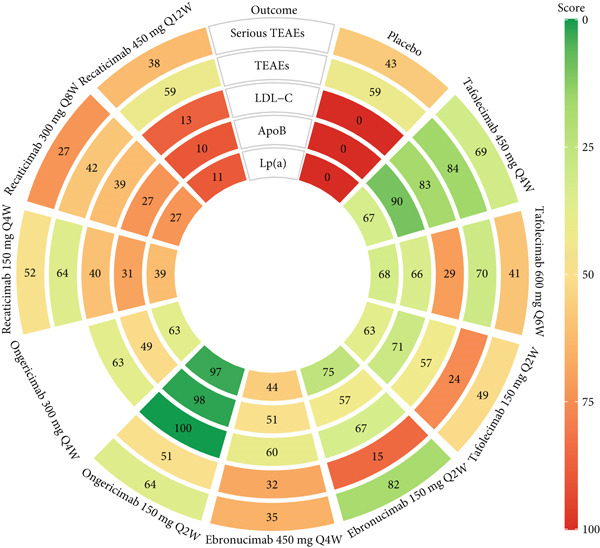
Rank‐heat plot with *P*‐scores. High‐ranking interventions are represented by darker green colors, whereas low‐ranking interventions are represented by darker red colors. Abbreviations: ApoB, apolipoprotein B; LDL‐C, low‐density lipoprotein cholesterol; Lp(a), lipoprotein (a); Q2, 4, 6, 8, 12 W, every 2, 4, 6, 8, 12 weeks; TEAEs, treatment‐emergent adverse events.

The NMA results for each efficacy outcome are systematically presented in Figure 3, with corresponding *P*‐score rankings detailed in Figure 4. Table S7 presents the *p* values adjusted for multiple comparisons using the Holm–Bonferroni method. The results demonstrated statistically significant improvements in lipid parameters across all agents′ dosing regimens versus placebo (adjusted‐*p* < 0.001).

Figure 3Network geometry and league table of (a) low‐density lipoprotein cholesterol, percentage; (b) apolipoprotein B, percentage; (c) lipoprotein (a), percentage. The upper right panel shows the corresponding network geometry of each outcome. The lower left panel shows the corresponding league table of each outcome. The results are represented in mean differences (MDs) and 95% confidence intervals (CIs). Shades in green mean “high” confidence rating; in blue mean “moderate” confidence rating; in yellow mean “low” confidence rating; in red mean “very low” confidence rating. Abbreviations: Q2, 4, 6, 8, 12 W, every 2, 4, 6, 8, 12 weeks. The comparisons should be read from right to left. The differences reported in the text, which are presented to clarify the direction of comparison, correspond to the inverse numerical values. For example, regarding ongericimab 150 mg Q2W versus placebo in terms of LDL‐C reduction, the text presents the following values: MD = −74.21*%*, 95% CI: −79.69% to −68.73%, which correspond to the differences shown in the figure: MD = 74.21*%*, 95% CI: 68.73%–79.69%.

**Figure:**
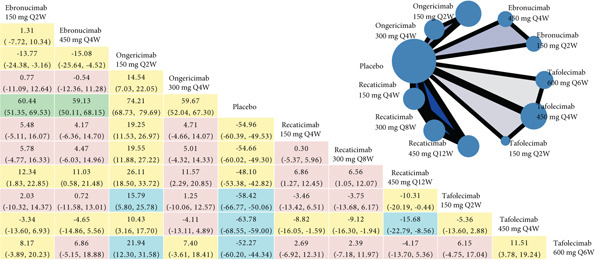
(a)

**Figure:**
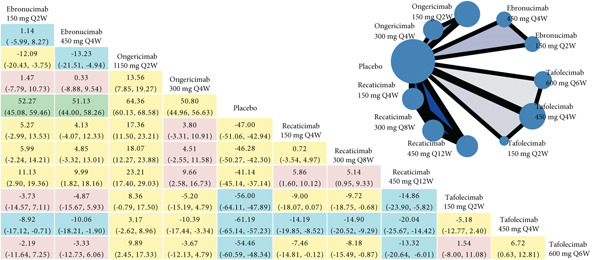
(b)

**Figure:**
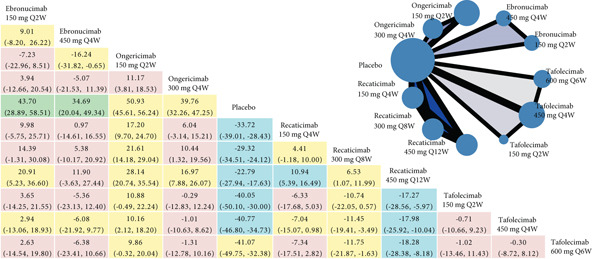
(c)

For LDL‐C reduction (Figure 3a), ongericimab 150 mg Q2W significantly reduced LDL‐C versus placebo (MD = −74.21*%*; 95% CI: −79.69% to −68.73%; adjusted‐*p* < 0.001) and showed significant absolute percentage differences compared with all active comparators (adjusted‐*p* < 0.05), but not with tafolecimab 150 mg Q2W (MD = −15.79*%*; 95% CI: −25.78% to −5.80%; adjusted‐*p* = 0.076) or 450 mg Q4W (MD = −10.43*%*; 95% CI: −17.70% to –3.16%; adjusted‐*p* = 0.182). The second‐ranked intervention, tafolecimab 450 mg Q4W, showed statistically significant superiority over recaticimab 450 mg Q12W (MD = −15.68*%*; 95% CI: –22.79% to –8.56%; adjusted‐*p* = 0.001). Recaticimab 450 mg Q12W demonstrated the least potent LDL‐C reduction versus placebo (MD = −48.10*%*; 95% CI: −53.38% to −42.82%; adjusted‐*p* < 0.001), ranking last in therapeutic efficacy. All remaining comparisons indicated therapeutic equivalence (adjusted‐*p* > 0.05).

In both ApoB and Lp(a) reduction profiles (Figures 3b and 3c), ongericimab 150 mg Q2W demonstrated sustained therapeutic superiority, achieving significant reductions versus placebo for ApoB (MD: −64.36%; 95% CI: −68.58% to −60.13%; adjusted‐*p* < 0.001) and Lp(a) (MD: −50.93%; 95% CI: −56.24% to −45.61%; adjusted‐P <0.001). For ApoB, ongericimab 150 mg Q2W outperformed ongericimab 300 mg Q4W and all recaticimab regimens (adjusted‐P <0.05), whereas the second‐ranked tafolecimab 450 mg Q4W showed significant advantages over all recaticimab doses (adjusted‐*p* < 0.05). In contrast, recaticimab 450 mg Q12W ranked lowest in ApoB efficacy, exhibiting inferiority to all tafolecimab regimens (adjusted‐*p* < 0.05) but still superiority to placebo (MD: −41.14%; 95% CI: −45.14% to −37.14%; adjusted‐*p* < 0.001). For Lp(a), ongericimab 150 mg Q2W significantly surpassed all recaticimab regimens (adjusted‐*p* < 0.05), whereas recaticimab 450 mg Q12W maintained the lowest ranking, with inferior reductions compared with ongericimab 300 mg Q4W, recaticimab 150 mg Q4W, tafolecimab 450 mg Q4W, and tafolecimab 600 mg Q6W (adjusted‐*p* < 0.05), despite outperforming placebo (MD: −22.79%; 95% CI: −27.94% to −17.63%; adjusted‐*p* < 0.001).

### 3.4. Network Estimates for Clinical Safety

Figure S6 presents the NMA results for safety profile. The investigation showed comparable incidence rates of TEAEs or serious TEAEs across all active treatment regimens (adjusted‐*p* > 0.999), with no evidence of dose‐dependent safety signal escalation. Meanwhile, none of the therapeutic interventions exhibited statistically significant elevation relative to placebo (adjusted‐*p* > 0.999). *P*‐score rankings in Figure 4 identified tafolecimab 450 mg Q4W as demonstrating the most favorable safety profile among interventions.

### 3.5. Covariate and Sensitivity Analyses

Substantial heterogeneity was detected for both LDL‐C and TEAEs outcomes, with *I* ^2^ statistics of 56.7% and 54.4%, respectively. The quasi‐Monte Carlo integration model demonstrated satisfactory goodness‐of‐fit (Figure S7), whereas meta‐regression analysis failed to identify significant moderators of heterogeneity (Figures S8 and S9). Random‐effects subgroup analysis revealed a trend toward interaction between baseline LDL‐C levels and LDL‐C‐lowering efficacy (Figure S10; *p* = 0.09), though no significant associations emerged between predefined covariates and TEAEs (Figure S11). Notably, the CREDIT‐4 trial was identified as an influential outlier in TEAEs outcome (RR = 0.77, 95% CI: 0.56–0.98). Exclusion of this trial resulted in complete resolution of heterogeneity (*τ* ^2^ = 0; *I* ^2^ = 0*%*).

Sensitivity analysis excluding high‐risk bias studies confirmed robustness of efficacy results (Figure S12). The absence of detailed safety events reporting in two high‐risk studies, which were already excluded from the main analysis, precluded conducting the sensitivity analysis.

### 3.6. TSA

Given the substantial number of treatment comparisons and outcome measures included in the NMA, TSA was applied to the most notable comparisons—specifically, ongericimab 150 mg Q2W versus placebo and tafolecimab 450 mg Q4W versus placebo—focusing on the key efficacy outcome (LDL‐C reduction) and safety outcome (TEAEs). This selective approach balanced methodological rigor with practical feasibility.

For LDL‐C reduction, the cumulative sample size of included studies substantially exceeded the diversity‐adjusted RIS. The first information fraction surpassed 100% of RIS, indicating that the current evidence base is sufficiently large to reliably detect the treatment effect (Figure S13). The TSA for TEAEs revealed a stark contrast (Figure S14). The diversity‐adjusted RIS for the ongericimab 150 mg Q2W comparison was 20,771 participants, yet only 1.23% of this target has been accrued to date. Similarly, the RIS for the tafolecimab 450 mg Q4W comparison was considerably larger, with only 2.02% accumulated. Furthermore, the cumulative *Z*‐curve did not cross any monitoring boundaries, suggesting the current evidence remains insufficient to draw firm conclusions regarding safety.

### 3.7. Certainty of Evidence

Figures 3 and S6 classified by color simply present the evidence ratings of each comparison, and additional information for each outcome is listed in Tables S8, S9, S10, S11, and S12, respectively. It is worth noting that most comparisons in the safety outcomes had “very low” levels of evidence, mainly due to the large range of effect size estimates.

## 4. Discussion

In summary, synthesizing efficacy metrics and safety rankings, all four PCSK9 monoclonal antibodies provide new options for the treatment of hypercholesterolemia. Ongericimab 150 mg Q2W is expected to be the preferred therapeutic strategy in lipid control among evaluated interventions; but given the frequency of administration and potential tolerability concerns, tafolecimab with the Q4W dosing schedule represents the optimal risk‐benefit equilibrium. While tafolecimab, ebronucimab, ongericimab, and recaticimab currently hold marketing authorization exclusively in China, this comprehensive NMA provides critical pharmacotherapeutic evidence supporting their potential utility across genetically and phenotypically analogous populations.

Currently, no direct RCTs or real‐world studies compare these agents with evolocumab or alirocumab. Drawing upon the currently available evidence, we propose a reasonable hypothesis that, within the Chinese population, alirocumab 75/150 mg Q2W may have inferior lipid‐lowering efficacy compared with ongericimab 150 mg Q2W, but potentially similar efficacy to tafolecimab 150 mg Q2W [39]. In contrast, evolocumab appears to exhibit greater efficacy regardless of dosing regimen (140 mg Q2W or 420 mg monthly), with its effect potentially comparable with either ongericimab 150 mg Q2W or tafolecimab 450 mg Q4W [40, 41]. This hypothesis of observed interethnic similarity in treatment response is derived from current data and warrants confirmation through well‐designed head‐to‐head RCTs. Direct annual maintenance costs derived from the catalog of Chinese medical insurance revealed lower expenditures for evolocumab 420 mg monthly (CNY 10,216.8) compared with tafolecimab 450 mg Q4W (CNY 11,154.0), with a cost difference of −CNY 937.2. While evolocumab 420 mg monthly may represent a cost‐effective PCSK9 inhibitor option for Chinese hypercholesterolemia patients, further rigorous cost‐effectiveness analyses are warranted as well [42]. Although recaticimab exhibits attenuated efficacy relative to other agents, its extended‐interval dosing regimen (12‐week administration) may enhance treatment adherence and reduce injection frequency and associated costs, particularly in developing countries [43]. Conversely, inclisiran, an ultra‐long‐acting small interfering RNA therapy, has limited accessibility due to prohibitive price, despite its biannual dosing schedule.

This investigation has several methodological constraints requiring cautious interpretation. First, the paucity of eligible RCTs investigating novel PCSK9 inhibitors currently approved only in China introduces potential selection bias and restricts analytical robustness. The limited number of included trials (*n* < 10) substantially constrains the validity of regression modeling and precludes formal evaluation of publication bias through funnel plots or Egger′s test. Furthermore, TSA indicates insufficient statistical power to detect clinically significant safety differences, precluding definitive conclusions. The long‐term safety of these medications also remains an unresolved issue that necessitates further investigation. While there are no considerable concerns regarding heterogeneity, it remains a factor that could substantially influence the results, as indicated by the evidence rating. Additionally, the exclusive enrollment of Chinese participants challenges external validity, particularly regarding pharmacokinetic and safety profile generalizability to other ethnic populations. Finally, though evolocumab and alirocumab demonstrated proven cardiovascular risk reduction in multiethnic cohorts [44–46], the cardiovascular benefit profiles of these novel agents remain undetermined. Large‐scale, ethnically diverse RCTs with extended double‐blind phases are imperative to address these limitations comprehensively.

## 5. Conclusion

These findings supported that all four PCSK9 monoclonal antibodies provided new options for the treatment of hypercholesterolemia and positioned ongericimab 150 mg Q2W as a clinically optimal PCSK9 inhibitor with robust lipid‐lowering capacity. The results highlight the potential of next‐generation PCSK9 monoclonal antibodies, particularly in East Asian populations, while underscoring the need for multinational trials to validate ethnic‐specific responses.

## Ethics Statement

This article is based on previously conducted studies and does not contain any new data with human participants or animals performed by any of the authors.

## Disclosure

All authors critically reviewed the report and approved the final draft.

## Conflicts of Interest

The authors declare no conflicts of interest.

## Author Contributions

S.W. designed the study, drafted the manuscript and figures. S.W., C.L., and D.F. contributed to the literature search, literature selection, data extraction, statistical analysis, and quality assessments.

## Funding

No funding was received for this manuscript.

## Supporting information


**Supporting Information 1** Additional supporting information can be found online in the Supporting Information section. Table S1: PRISMA 2020 checklist. Table S2: PICOS study selection criteria. Table S3: Search strategy. Table S4: Clinical efficacy data for NMA. Table S5: Clinical safety data for NMA. Table S6: Tests of heterogeneity and inconsistency. Table S7: *p* values adjusted for multiple comparisons using the Holm–Bonferroni method. Table S8: The certainty of evidence in LDL‐C outcome. Table S9: The certainty of evidence in ApoB outcome. Table S10: The certainty of evidence in Lp(a) outcome. Table S11: The certainty of evidence in TEAEs outcome. Table S12: The certainty of evidence in serious TEAEs outcome. Figure S1: The forest plot of direct and indirect evidence for estimating the percentage change in LDL‐C, percentage. Figure S2: The forest plot of direct and indirect evidence for estimating the percentage change in ApoB, percentage. Figure S3: The forest plot of direct and indirect evidence for estimating the percentage change in Lp(a), percentage. Figure S4: The forest plot of direct and indirect evidence for estimating the risk ratio of TEAEs. Figure S5: The forest plot of direct and indirect evidence for estimating the risk ratio of serious TEAEs. Figure S6: Network geometry and league table of (A) TEAEs; (B) serious TEAEs. Figure S7: Residual deviance contributions of (A) LDL‐C outcome in random‐effects model; (B) TEAEs outcome in fixed‐effects model. Figure S8: Meta‐regression analysis of clinical efficacy in LDL‐C outcome. Figure S9: Meta‐regression analysis of clinical safety in TEAEs outcome. Figure S10: Subgroup analysis of PCSK9 inhibitors versus placebo in LDL‐C outcome, percentage. Figure S11: Subgroup analysis of PCSK9 inhibitors versus placebo in TEAEs outcome. Figure S12. Sensitivity analysis of clinical efficacy by excluding high‐risk studies. Figure S13: Trial sequential analysis of LDL‐C percentage reduction. Figure S14: Trial sequential analysis of TEAEs incidence.

## Data Availability

The data that supports the findings of this study is available in the supporting information of this article.
