# Supplementary material for: The Efficacy and Safety of Four Novel PCSK9 Monoclonal Antibodies in Patients With Hypercholesterolemia: A Systematic Review With Network Meta‐Analysis and Trial Sequential Analysis
Source: Cardiovasc Ther. 2026 Jan 24;2026:6345873. doi: 10.1155/cdr/6345873 (PMC12831481; doi:10.1155/cdr/6345873)
Supplement: Supplementary file 1 — Supporting Information 1 Additional supporting information can be found online in the Supporting Information section. Table S1: PRISMA 2020 checklist. Table S2: PICOS study selection criteria. Table S3: Search strategy. Table S4: Clinical efficacy data for NMA. Table S5: Clinical safety data for NMA. Table S6: Tests of heterogeneity and inconsistency. Table S7: p values adjusted for multiple comparisons using the Holm–Bonferroni method. Table S8: The certainty of evidence in LDL‐C outcome. Table S9: The certainty of evidence in ApoB outcome. Table S10: The certainty of evidence in Lp(a) outcome. Table S11: The certainty of evidence in TEAEs outcome. Table S12: The certainty of evidence in serious TEAEs outcome. Figure S1: The forest plot of direct and indirect evidence for estimating the percentage change in LDL‐C, percentage. Figure S2: The forest plot of direct and indirect evidence for estimating the percentage change in ApoB, percentage. Figure S3: The forest plot of direct and indirect evidence for estimating the percentage change in Lp(a), percentage. Figure S4: The forest plot of direct and indirect evidence for estimating the risk ratio of TEAEs. Figure S5: The forest plot of direct and indirect evidence for estimating the risk ratio of serious TEAEs. Figure S6: Network geometry and league table of (A) TEAEs; (B) serious TEAEs. Figure S7: Residual deviance contributions of (A) LDL‐C outcome in random‐effects model; (B) TEAEs outcome in fixed‐effects model. Figure S8: Meta‐regression analysis of clinical efficacy in LDL‐C outcome. Figure S9: Meta‐regression analysis of clinical safety in TEAEs outcome. Figure S10: Subgroup analysis of PCSK9 inhibitors versus placebo in LDL‐C outcome, percentage. Figure S11: Subgroup analysis of PCSK9 inhibitors versus placebo in TEAEs outcome. Figure S12. Sensitivity analysis of clinical efficacy by excluding high‐risk studies. Figure S13: Trial sequential analysis of LDL‐C percentage reduction. Figure S14: Trial sequential a [file CDR-2026-6345873-s001.docx]

**Supporting Information**

**The Efficacy and Safety of Four Novel PCSK9 Monoclonal Antibodies in Patients with Hypercholesterolemia: A Systematic Review with Network Meta-analysis and Trial Sequential Analysis**

Sihua Wang^1^, Chenyu Li^2^, Duncong Fan^1,*^

^1^Department of Pharmacy, Haining People’s Hospital (The Affiliated Haining Hospital of Jiaxing University), No.2 Qianjiang West Road, Haining, Zhejiang 314400, China

^2^Department of Pharmacy, Haining Hospital of Traditional Chinese Medicine, No.177 Changdai Road, Haining, Zhejiang 314400, China

*Corresponding author, email: hnfanfan1983@163.com

Contents

[**Table S1.** PRISMA 2020 checklist. 4](#_Toc203847358)

[**Table S2.** PICOS study selection criteria. 6](#_Toc203847359)

[**Table S3.** Search strategy. 7](#_Toc203847360)

[**Table S4.** Clinical efficacy data for NMA. 9](#_Toc203847361)

[**Table S5.** Clinical safety data for NMA. 10](#_Toc203847362)

[**Table S6.** Tests of heterogeneity and inconsistency. 11](#_Toc203847363)

[**Table S7.** P-values adjusted for multiple comparisons using the Holm-Bonferroni method. 12](#_Toc203847364)

[**Table S8.** The certainty of evidence in LDL-C outcome. 15](#_Toc203847365)

[**Table S9.** The certainty of evidence in ApoB outcome. 17](#_Toc203847366)

[**Table S10.** The certainty of evidence in Lp(a) outcome. 19](#_Toc203847367)

[**Table S11.** The certainty of evidence in TEAEs outcome. 21](#_Toc203847368)

[**Table S12.** The certainty of evidence in serious TEAEs outcome. 23](#_Toc203847369)

[**Figure S1.** The forest plot of direct and indirect evidence for estimating the percentage change in LDL-C, %. 25](#_Toc203847370)

[**Figure S2.** The forest plot of direct and indirect evidence for estimating the percentage change in ApoB, %. 26](#_Toc203847371)

[**Figure S3.** The forest plot of direct and indirect evidence for estimating the percentage change in Lp(a), %. 27](#_Toc203847372)

[**Figure S4.** The forest plot of direct and indirect evidence for estimating the risk ratio of TEAEs. 28](#_Toc203847373)

[**Figure S5.** The forest plot of direct and indirect evidence for estimating the risk ratio of serious TEAEs. 29](#_Toc203847374)

[**Figure S6.** Network geometry and league table of **(A)** TEAEs; **(B)** serious TEAEs. 30](#_Toc203847375)

[**Figure S7.** Residual deviance contributions of **(A)** LDL-C outcome in random-effects model; **(B)** TEAEs outcome in fixed-effects model. 31](#_Toc203847376)

[**Figure S8.** Meta-regression analysis of clinical efficacy in LDL-C outcome. 32](#_Toc203847377)

[**Figure S9.** Meta-regression analysis of clinical safety in TEAEs outcome. 33](#_Toc203847378)

[**Figure S10.** Subgroup analysis of PCSK9 inhibitors versus placebo in LDL-C outcome, %. 34](#_Toc203847379)

[**Figure S11.** Subgroup analysis of PCSK9 inhibitors versus placebo in TEAEs outcome. 35](#_Toc203847380)

[**Figure S12.** Sensitivity analysis of clinical efficacy by excluding high-risk studies. 36](#_Toc203847381)

[**Figure S13.** Trial sequential analysis of LDL-C percentage reduction. 37](#_Toc203847382)

[**Figure S14.** Trial sequential analysis of TEAEs incidence. 38](#_Toc203847383)

**Table S1.** PRISMA 2020 checklist.

| **Section and Topic** | **Item #** | **Checklist item** | **Location where item is reported** |
| --- | --- | --- | --- |
| **TITLE** | | |  |
| Title | 1 | Identify the report as a systematic review. | Draft P1 |
| **ABSTRACT** | | |  |
| Abstract | 2 | See the PRISMA 2020 for Abstracts checklist. | Draft P1 |
| **INTRODUCTION** | | |  |
| Rationale | 3 | Describe the rationale for the review in the context of existing knowledge. | Draft P1-P2 |
| Objectives | 4 | Provide an explicit statement of the objective(s) or question(s) the review addresses. | Draft P2 |
| **METHODS** | | |  |
| Eligibility criteria | 5 | Specify the inclusion and exclusion criteria for the review and how studies were grouped for the syntheses. | Draft P2-P3 |
| Information sources | 6 | Specify all databases, registers, websites, organisations, reference lists and other sources searched or consulted to identify studies. Specify the date when each source was last searched or consulted. | Draft P3 |
| Search strategy | 7 | Present the full search strategies for all databases, registers and websites, including any filters and limits used. | Table S2 |
| Selection process | 8 | Specify the methods used to decide whether a study met the inclusion criteria of the review, including how many reviewers screened each record and each report retrieved, whether they worked independently, and if applicable, details of automation tools used in the process. | Draft P3 |
| Data collection process | 9 | Specify the methods used to collect data from reports, including how many reviewers collected data from each report, whether they worked independently, any processes for obtaining or confirming data from study investigators, and if applicable, details of automation tools used in the process. | Draft P3 |
| Data items | 10a | List and define all outcomes for which data were sought. Specify whether all results that were compatible with each outcome domain in each study were sought (e.g. for all measures, time points, analyses), and if not, the methods used to decide which results to collect. | Draft P3 |
|  | 10b | List and define all other variables for which data were sought (e.g. participant and intervention characteristics, funding sources). Describe any assumptions made about any missing or unclear information. | Draft P3 |
| Study risk of bias assessment | 11 | Specify the methods used to assess risk of bias in the included studies, including details of the tool(s) used, how many reviewers assessed each study and whether they worked independently, and if applicable, details of automation tools used in the process. | Draft P3 |
| Effect measures | 12 | Specify for each outcome the effect measure(s) (e.g. risk ratio, mean difference) used in the synthesis or presentation of results. | Draft P3 |
| Synthesis methods | 13a | Describe the processes used to decide which studies were eligible for each synthesis (e.g. tabulating the study intervention characteristics and comparing against the planned groups for each synthesis (item #5)). | Draft P2 |
|  | 13b | Describe any methods required to prepare the data for presentation or synthesis, such as handling of missing summary statistics, or data conversions. | Draft P4 |
|  | 13c | Describe any methods used to tabulate or visually display results of individual studies and syntheses. | Draft P4 |
|  | 13d | Describe any methods used to synthesize results and provide a rationale for the choice(s). If meta-analysis was performed, describe the model(s), method(s) to identify the presence and extent of statistical heterogeneity, and software package(s) used. | Draft P4 |
|  | 13e | Describe any methods used to explore possible causes of heterogeneity among study results (e.g. subgroup analysis, meta-regression). | Draft P4 |
|  | 13f | Describe any sensitivity analyses conducted to assess robustness of the synthesized results. | Draft P4 |
| Reporting bias assessment | 14 | Describe any methods used to assess risk of bias due to missing results in a synthesis (arising from reporting biases). | Draft P4 |
| Certainty assessment | 15 | Describe any methods used to assess certainty (or confidence) in the body of evidence for an outcome. | Draft P4 |
| **RESULTS** | | |  |
| Study selection | 16a | Describe the results of the search and selection process, from the number of records identified in the search to the number of studies included in the review, ideally using a flow diagram. | Draft P4 (Fig. 1) |
|  | 16b | Cite studies that might appear to meet the inclusion criteria, but which were excluded, and explain why they were excluded. | Draft P4 |
| Study characteristics | 17 | Cite each included study and present its characteristics. | Table 1 |
| Risk of bias in studies | 18 | Present assessments of risk of bias for each included study. | Figure 2 |
| Results of individual studies | 19 | For all outcomes, present, for each study: (a) summary statistics for each group (where appropriate) and (b) an effect estimate and its precision (e.g. confidence/credible interval), ideally using structured tables or plots. | Table S4, and S5 |
| Results of syntheses | 20a | For each synthesis, briefly summarise the characteristics and risk of bias among contributing studies. | Draft P5 |
|  | 20b | Present results of all statistical syntheses conducted. If meta-analysis was done, present for each the summary estimate and its precision (e.g. confidence/credible interval) and measures of statistical heterogeneity. If comparing groups, describe the direction of the effect. | Draft P5, P6, and Fig. 3, Fig. S6 |
|  | 20c | Present results of all investigations of possible causes of heterogeneity among study results. | Draft P6 |
|  | 20d | Present results of all sensitivity analyses conducted to assess the robustness of the synthesized results. | Draft P6 |
| Reporting biases | 21 | Present assessments of risk of bias due to missing results (arising from reporting biases) for each synthesis assessed. | NA |
| Certainty of evidence | 22 | Present assessments of certainty (or confidence) in the body of evidence for each outcome assessed. | Draft P6 |
| **DISCUSSION** | | |  |
| Discussion | 23a | Provide a general interpretation of the results in the context of other evidence. | Draft P7 |
|  | 23b | Discuss any limitations of the evidence included in the review. | Draft P7 |
|  | 23c | Discuss any limitations of the review processes used. | Draft P7 |
|  | 23d | Discuss implications of the results for practice, policy, and future research. | Draft P7-8 |
| **OTHER INFORMATION** | | |  |
| Registration and protocol | 24a | Provide registration information for the review, including register name and registration number, or state that the review was not registered. | Draft P2 |
|  | 24b | Indicate where the review protocol can be accessed, or state that a protocol was not prepared. | Draft P2 |
|  | 24c | Describe and explain any amendments to information provided at registration or in the protocol. | NA |
| Support | 25 | Describe sources of financial or non-financial support for the review, and the role of the funders or sponsors in the review. | Draft P8 |
| Competing interests | 26 | Declare any competing interests of review authors. | Draft P8 |
| Availability of data, code and other materials | 27 | Report which of the following are publicly available and where they can be found: template data collection forms; data extracted from included studies; data used for all analyses; analytic code; any other materials used in the review. | Draft P8 |

**Table S2.** PICOS study selection criteria.

| Criteria | Description | |
| --- | --- | --- |
| Population | Patients with hypercholesterolemia | |
| Interventions | - Tafolecimab - Ebronucimab - Ongericimab - Recaticimab | |
| Comparator | Placebo | |
| Outcomes | *Efficacy*   - The percentage change from baseline of low-density lipoprotein cholesterol (LDL-C) - The percentage change from baseline of apolipoprotein B (ApoB) - The percentage change from baseline of lipoprotein (a) (Lp[a]) | *Safety*   - The incidence of treatment-emergent adverse events (TEAEs) - The incidence of serious TEAEs |
| Study design | Phase 3 randomized controlled trials | |
| Language | No language restriction | |
| Abbreviations: PICOS, population, intervention, comparator, outcome, study design. | | |

| **Table S3.** Search strategy. | | |
| --- | --- | --- |
| No. | Terms | Hits |
| Database: PubMed  Date of search: Feburary 1, 2025 | | |
| #1 | "RandomizedControlled Trial"[Publication Type] OR "RandomizedControlled Trials as Topic"[MeSH Terms] OR "randomized"[Title/Abstract]OR "controlled"[Title/Abstract]OR "trial"[Title/Abstract]OR "phase III"[Title/Abstract]OR "phase 3"[Title/Abstract] | 2,097,513 |
| #2 | (tafolecimab) OR (recaticimab) OR (IBI-306) OR (IBI306) OR (SHR-1209) OR (SHR1209) OR (sintbilo) OR (ebronucimab) OR (AK-102) OR (AK102) OR (ongericimab) OR (JS002) OR (JS-002) | 28 |
| #3 | "Hypercholesterolemia"[Mesh] OR"Hypercholesterolemia"[Title/Abstract] OR"Hypercholesterolemias"[Title/Abstract] OR"Hypercholesteremia"[Title/Abstract] OR"Hypercholesteremias"[Title/Abstract] OR"Elevated Cholesterol"[Title/Abstract] OR"Cholesterol, Elevated"[Title/Abstract] OR"Elevated Cholesterols"[Title/Abstract] OR"High Cholesterol Levels"[Title/Abstract] OR"Cholesterol Level, High"[Title/Abstract] OR"Cholesterol Levels, High"[Title/Abstract] OR"High Cholesterol Level"[Title/Abstract] OR"Level, High Cholesterol"[Title/Abstract] OR"non-familial"[Title/Abstract] OR"familial"[Title/Abstract] OR"heterozygous"[Title/Abstract] OR"homozygous"[Title/Abstract] OR"primary"[Title/Abstract] OR ”secondary” [Title/Abstract] OR "polygenic"[Title/Abstract] OR “mixed hyperlipidemia"[Title/Abstract] | 2,988,176 |
| #4 | #1 AND #2 AND #3 | 14 |
| Database: Cochrane Library  Date of search: Feburary 1, 2025 | | |
| #1 | MeSH descriptor: [Clinical Trials as Topic] explode all trees | 99,842 |
| #2 | ("randomised" OR “randomized” OR "controlled" OR "phase 3" OR "phase III" OR "trial"):ti,ab,kw | 1,782,124 |
| #3 | ("recaticimab" OR "IBI-306" OR "IBI306" OR "tafolecimab" OR "SHR-1209" OR "SHR1209" OR "sintbilo"OR “ebronucimab” OR “AK-102” OR “AK102” OR “ongericimab” OR “JS002” OR “JS-002”) | 58 |
| #4 | MeSH descriptor: [Hypercholesterolemia] explode all trees | 4,314 |
| #5 | MeSH descriptor: [Hyperlipoproteinemia Type II] explode all trees | 678 |
| #6 | MeSH descriptor: [Hyperlipoproteinemia Type III] explode all trees | 24 |
| #7 | ("hypercholesterolemia" OR "hypercholesterolemias" OR "hypercholesteremia" OR "hypercholesteremias" OR "non-familial" OR"familial" OR"heterozygous" OR"homozygous" OR"primary" OR ”secondary” OR "polygenic" OR "mixed hyperlipidemia"):ti,ab,kw | 738,203 |
| #8 | (#1 OR #2) AND #3 AND (#4 OR #5 OR #6 OR #7) | 43 |
| Database: Embase  Date of search: Feburary 1, 2025 | | |
| #1 | ('hypercholesterolemia'/exp OR 'hypercholesterolemia' OR 'hypercholesterolemias' OR 'hypercholesteremia'/exp OR 'hypercholesteremia' OR 'hypercholesteremias' OR 'non-familial' OR 'familial' OR 'heterozygous' OR 'homozygous' OR 'primary' OR 'secondary'/exp OR 'secondary' OR 'polygenic' OR 'mixed hyperlipidemia'/exp OR 'mixed hyperlipidemia') AND [01-01-1000]/sd NOT [02-02-2025]/sd | 4,537,442 |
| #2 | [controlled clinical trial]/lim OR [randomized controlled trial]/lim AND [01-01-1000]/sd NOT [02-02-2025]/sd | 1,046,678 |
| #3 | 'recaticimab'/exp OR 'tafolecimab'/exp OR 'ibi-306' OR 'ibi306' OR 'shr-1209' OR 'shr1209' OR 'sintbilo' OR ‘ebronucimab’/exp OR ‘AK-102’ OR ‘AK102’ OR ‘ongericimab’/exp OR ‘JS002’ OR ‘JS-002’ AND [01-01-1000]/sd NOT [02-02-2025]/sd | 50 |
| #4 | #1 AND #2 AND #3 | 18 |
| Database: Web of Science  Date of search: Feburary 1, 2025 | | |
| #1 | ((((((TS=(Randomized Controlled Trial)) OR TS=(randomized)) OR TS=(controlled)) OR TS=(trial)) OR TS=(phase III)) OR TS=(phase 3)) OR TS=(randomised) | 26,888,733 |
| #2 | (((((((((((((TS=(tafolecimab)) OR TS=(IBI-306)) OR TS=(IBI306)) OR TS=(recaticimab)) OR TS=(SHR-1209)) OR TS=(SHR1209)) OR TS=(sintbilo)) OR TS=(ebronucimab)) OR TS=(AK-102)) OR TS=(AK102)) OR TS=(ongericimab)) OR TS=(JS002)) OR TS=(JS-002)) | 53 |
| #3 | (((((((((((TS=(hypercholesterolemia)) OR TS=(hypercholesterolemias)) OR TS=(hypercholesteremia)) OR TS=(hypercholesteremias)) OR TS=(non-familial)) OR TS=(familial) OR TS=(heterozygous)) OR TS=(homozygous))ORTS=(primary)) OR TS=(secondary)) OR TS=(polygenic)) OR TS=(mixed hyperlipidemia)) | 6,769,513 |
| #4 | #1 AND #2 AND #3  Refined By: Not Documents Type: Abstract or Review Article or Meeting | 13 |
| Register: ClinicalTrials.gov  Date of search: Feburary 1, 2025 | | |
| #1 | ("tafolecimab" OR "IBI-306" OR "IBI306" OR "recaticimab" OR "SHR1209" OR "SHR-1209" OR "sintbilo" OR “ebronucimab” OR “AK-102” OR “AK102” OR “ongericimab” OR “JS002” OR “JS-002”) AND ("Hypercholesterolemia"OR "Hypercholesterolemias"OR "Hypercholesteremia" OR "Hypercholesteremias" OR "Elevated Cholesterol"OR "Cholesterol, Elevated" OR "Elevated Cholesterols" OR "High Cholesterol Levels"OR "Cholesterol Level, High" OR "Cholesterol Levels, High" OR "High Cholesterol Level" OR "Level, High Cholesterol" OR "non-familial" OR "familial" OR "heterozygous" OR "homozygous"OR "primary" OR ”secondary” OR "polygenic"OR "mixed hyperlipidemia") | 35 |

| **Table S4.** Clinical efficacy data for NMA. | | | | | | | | | | | | | | | |
| --- | --- | --- | --- | --- | --- | --- | --- | --- | --- | --- | --- | --- | --- | --- | --- |
| Study | | Percentage CFB in LDL-C | | | | Percentage CFB in ApoB | | | | | Percentage CFB in Lp(a) | | | | |
|  |  | Mean, % | SD | Pantients, n | | Mean, % | | SD | Pantients, n | | Mean, % | | SD | Pantients, n | |
| Huo et al. 2023 (CREDIT-1) | |  |  |  | |  | |  |  | |  | |  |  | |
| Placebo | | 3.55 | 30.27 | 200 | | 5.51 | | 23.6 | 199 | | 18.86 | | 43.42 | 199 | |
| TAFO 450 mg Q4W | | -62.3 | 21.78 | 206 | | -58.5 | | 22.91 | 205 | | -28.3 | | 37.23 | 205 | |
| TAFO 600 mg Q6W | | -49.9 | 34.17 | 202 | | -50.4 | | 27.93 | 195 | | -25.8 | | 40.5 | 195 | |
| Chai et al. 2023 (CREDIT-2) | |  |  |  | |  | |  |  | |  | |  |  | |
| Placebo | | 2.72 | 20.07 | 48 | | 4.09 | | 34.01 | 48 | | -1.61 | | 28.28 | 48 | |
| TAFO 150 mg Q2W | | -54.67 | 17.26 | 52 | | -51.09 | | 18.07 | 52 | | -40.33 | | 24.55 | 52 | |
| TAFO 450 mg Q4W | | -59.16 | 17.59 | 48 | | -56.02 | | 16.86 | 48 | | -39.85 | | 26.53 | 48 | |
| Qi et al. 2023 (CREDIT-4) | |  |  |  | |  | |  |  | |  | |  |  | |
| Placebo | | -4.87 | 18.81 | 98 | | -2.45 | | 15.84 | 98 | | -11.45 | | 39 | 98 | |
| TAFO 450 mg Q4W | | -67.9 | 21.37 | 203 | | -61.4 | | 18.43 | 201 | | -45.38 | | 43.38 | 201 | |
| Zhang et al. 2024 | |  |  |  | |  | |  |  | |  | |  |  | |
| Placebo | | -5.77 | 30.44 | 153 | | -4.15 | | 26.20 | 153 | | 3.98 | | 48.12 | 153 | |
| EBRO 150 mg Q2W | | -66.21 | 30.47 | 153 | | -56.42 | | 26.26 | 153 | | -39.72 | | 74.54 | 153 | |
| EBRO 450 mg Q4W | | -64.90 | 29.89 | 155 | | -55.28 | | 25.71 | 155 | | -30.71 | | 73.74 | 155 | |
| Wang et al. 2024 | |  |  |  | |  | |  |  | |  | |  |  | |
| Placebo | | 5.75 | 28.92 | 265 | | 4.25 | | 24.66 | 265 | | -1.89 | | 34.70 | 265 | |
| ONGE 150 mg Q2W | | -70.5 | 24.82 | 272 | | -61.5 | | 21.04 | 272 | | -53.1 | | 31.13 | 272 | |
| ONGE 300 mg Q4W | | -55.0 | 34.88 | 265 | | -47.3 | | 29.07 | 265 | | -41.8 | | 40.70 | 265 | |
| Zhao et al. 2024 | |  |  |  | |  | |  |  | |  | |  |  | |
| Placebo | | -2.56 | 20.56 | 86 | | -2.95 | | 18.57 | 86 | | -2.93 | | 24.31 | 86 | |
| ONGE 150 mg Q2W | | -74.48 | 19.73 | 169 | | -65.58 | | 17.93 | 169 | | -53.53 | | 23.44 | 169 | |
| Xu et al. 2024 (REMAIN-1) | |  |  |  | |  | |  |  | |  | |  |  | |
| Placebo | | 0.31 | 16.92 | 235 | | 1.14 | | 13.71 | 235 | | -1.75 | | 28.61 | 235 | |
| RECA 150 mg Q4W | | -50.4 | 19.18 | 157 | | -43.5 | | 15.34 | 157 | | -35.1 | | 30.69 | 157 | |
| RECA 300 mg Q8W | | -50.5 | 16.57 | 156 | | -43.2 | | 13.7 | 156 | | -27 | | 28.36 | 156 | |
| RECA 450 mg Q12W | | -45.6 | 14.93 | 155 | | -39.9 | | 13.66 | 155 | | -22.5 | | 27.63 | 155 | |
| Sun et al. 2024 (REMAIN-2) | |  |  |  | |  | |  |  | |  | |  |  | |
| Placebo | | 6.18 | 27.83 | 233 | | 4.78 | | 24.27 | 233 | | -3.06 | | 27.34 | 233 | |
| RECA 150 mg Q4W | | -54.9 | 30.92 | 153 | | -46.3 | | 26.82 | 153 | | -37.2 | | 28.08 | 153 | |
| RECA 300 mg Q8W | | -54.2 | 31.03 | 151 | | -44.9 | | 26.02 | 151 | | -36.3 | | 27.27 | 151 | |
| RECA 450 mg Q12W | | -44.8 | 29.25 | 152 | | -36.1 | | 26.73 | 152 | | -27.8 | | 26.73 | 152 | |
| Abbreviations: ApoB, apolipoprotein B; CFB, change from baseline; EBRO, ebronucimab; LDL-C, low-density lipoprotein cholesterol; Lp(a), lipoprotein (a); ONGE, ongericimab; Q2, 4, 6, 8, 12W, every 2, 4, 6, 8, 12 weeks; SD, standard deviation; TAFO, tafolecimab; RECA, recaticimab. | | | | | | | | | | | | | | | |
| **Table S5.** Clinical safety data for NMA. | | | | | | | | | | | | | |  |  |
| Study | | | | TEAEs | | | | | Serious TEAEs | | | | |  |  |
|  |  |  |  | Participants occurred, n | | Total patients, n | | | Participants occurred, n | | Total patients, n | | |  |  |
| Huo et al. 2023 (CREDIT-1) | | | | | | | | | | | | | |  |  |
| Placebo | | | | 174 | | 203 | | | 21 | | 203 | | |  |  |
| Tafolecimab 450 mg Q4W | | | | 177 | | 209 | | | 15 | | 209 | | |  |  |
| Tafolecimab 600 mg Q6W | | | | 173 | | 202 | | | 21 | | 202 | | |  |  |
| Chai et al. 2023 (CREDIT-2) | | | | | | | | | | | | | |  |  |
| Placebo | | | | 28 | | 48 | | | 0 | | 48 | | |  |  |
| Tafolecimab 150 mg Q2W | | | | 36 | | 52 | | | 2 | | 52 | | |  |  |
| Tafolecimab 450 mg Q4W | | | | 23 | | 48 | | | 2 | | 48 | | |  |  |
| Qi et al. 2023 (CREDIT-4) | | | | | | | | | | | | | |  |  |
| Placebo | | | | 53 | | 98 | | | 4 | | 98 | | |  |  |
| Tafolecimab 450 mg Q4W | | | | 85 | | 205 | | | 5 | | 205 | | |  |  |
| Zhang et al. 2024 | | | | | | | | | | | | | |  |  |
| Placebo | | | | 57 | | 153 | | | 5 | | 153 | | |  |  |
| Ebronucimab 150 mg Q2W | | | | 75 | | 153 | | | 2 | | 153 | | |  |  |
| Ebronucimab 450 mg Q4W | | | | 68 | | 155 | | | 6 | | 155 | | |  |  |
| Wang et al. 2024^a^ | | | | | | | | | | | | | |  |  |
| Placebo | | | | 222 | | 265 | | | 36 | | 265 | | |  |  |
| Ongericimab 150 mg Q2W | | | | 418 | | 537 | | | 68 | | 537 | | |  |  |
| Ongericimab 300 mg Q4W | | | |  |  |  |  |  |  |  |  |  |  |  |  |
| Zhao et al. 2024 | | | | | | | | | | | | | |  |  |
| Placebo | | | | 38 | | 86 | | | 3 | | 85 | | |  |  |
| Ongericimab 150 mg Q2W | | | | 78 | | 169 | | | 4 | | 169 | | |  |  |
| Xu et al. 2024 (REMAIN-1)^b^ | | | | | | | | | | | | | |  |  |
| Placebo | | | | Not available | | | | | | | | | |  |  |
| Recaticimab 150 mg Q4W | | | |  |  |  |  |  |  |  |  |  |  |  |  |
| Recaticimab 300 mg Q8W | | | |  |  |  |  |  |  |  |  |  |  |  |  |
| Recaticimab 450 mg Q12W | | | |  |  |  |  |  |  |  |  |  |  |  |  |
| Sun et al. 2024 (REMAIN-2) | | | | | | | | | | | | | |  |  |
| Placebo | | | | 193 | | 233 | | | 20 | | 233 | | |  |  |
| Recaticimab 150 mg Q4W | | | | 124 | | 153 | | | 12 | | 153 | | |  |  |
| Recaticimab 300 mg Q8W | | | | 135 | | 151 | | | 16 | | 151 | | |  |  |
| Recaticimab 450 mg Q12W | | | | 126 | | 152 | | | 14 | | 152 | | |  |  |
| ^a^ The study would be excluded in network meta-analysis as it did not report data stratified by dose groups.  ^b^ The study only reported treatment-related adverse events.  Abbreviations: TEAEs, treatment-emergent adverse events; Q2, 4, 6, 8, 12W, every 2, 4, 6, 8, 12 weeks. | | | | | | | | | | | | | |  |  |

| **Table S6.** Tests of heterogeneity and inconsistency. | | | | | |
| --- | --- | --- | --- | --- | --- |
| Clinical outcomes | P-value | | | *I*^2^, % | τ^2^ |
|  | Within designs | Between designs | Total |  |  |
| LDL-C | 0.0097 | 0.4862 | 0.0313 | 56.7 | 9.3704 |
| ApoB | 0.0721 | 0.2969 | 0.0987 | 43.8 | 4.4728 |
| Lp(a) | 0.2261 | 0.2027 | 0.1759 | 33 | 5.5661 |
| TEAEs | -- | 0.1115 | 0.1115 | 54.4 | 0.0147 |
| Serious TEAEs | -- | 0.4306 | 0.4306 | 0 | 0 |
| Abbreviations: ApoB, apolipoprotein B; LDL-C, low-density lipoprotein cholesterol; Lp(a), lipoprotein (a); TEAEs, treatment-emergent adverse events. | | | | | |

| **Table S7.** P-values adjusted for multiple comparisons using the Holm-Bonferroni method. | | | | | | | | | | | |
| --- | --- | --- | --- | --- | --- | --- | --- | --- | --- | --- | --- |
| Comparisons | | LDL-C | | ApoB | | Lp(a) | | TEAEs | | Serious TEAEs | |
|  |  | P-value | Adjusted  P-value | P-value | Adjusted  P-value | P-value | Adjusted  P-value | P-value | Adjusted  P-value | P-value | Adjusted P-value |
| Ebronucimab 150 mg Q2W | Ebronucimab 450 mg Q4W | 0.776 | 1.000 | 0.754 | 1.000 | 0.305 | 1.000 | 0.520 | 1.000 | 0.179 | 1.000 |
| Ebronucimab 150 mg Q2W | Ongericimab 150 mg Q2W | 0.011 | 0.385 | 0.005 | 0.153 | 0.368 | 1.000 | 0.379 | 1.000 | 0.644 | 1.000 |
| Ebronucimab 450 mg Q4W | Ongericimab 150 mg Q2W | 0.005 | 0.184 | 0.002 | 0.063 | 0.041 | 1.000 | 0.651 | 1.000 | 0.553 | 1.000 |
| Ebronucimab 150 mg Q2W | Ongericimab 300 mg Q4W | 0.899 | 1.000 | 0.755 | 1.000 | 0.642 | 1.000 | - | - | - | - |
| Ebronucimab 450 mg Q4W | Ongericimab 300 mg Q4W | 0.929 | 1.000 | 0.944 | 1.000 | 0.546 | 1.000 | - | - | - | - |
| Ongericimab 150 mg Q2W | Ongericimab 300 mg Q4W | 0.000 | 0.006 | 0.000 | 0.000 | 0.003 | 0.109 | - | - | - | - |
| Ebronucimab 150 mg Q2W | Placebo | 0.000 | 0.000 | 0.000 | 0.000 | 0.000 | 0.000 | 0.128 | 1.000 | 0.269 | 1.000 |
| Ebronucimab 450 mg Q4W | Placebo | 0.000 | 0.000 | 0.000 | 0.000 | 0.000 | 0.000 | 0.375 | 1.000 | 0.776 | 1.000 |
| Ongericimab 150 mg Q2W | Placebo | 0.000 | 0.000 | 0.000 | 0.000 | 0.000 | 0.000 | 0.819 | 1.000 | 0.595 | 1.000 |
| Ongericimab 300 mg Q4W | Placebo | 0.000 | 0.000 | 0.000 | 0.000 | 0.000 | 0.000 | - | - | - | - |
| Ebronucimab 150 mg Q2W | Recaticimab 150 mg Q4W | 0.310 | 1.000 | 0.211 | 1.000 | 0.214 | 1.000 | 0.183 | 1.000 | 0.359 | 1.000 |
| Ebronucimab 450 mg Q4W | Recaticimab 150 mg Q4W | 0.438 | 1.000 | 0.324 | 1.000 | 0.903 | 1.000 | 0.412 | 1.000 | 0.707 | 1.000 |
| Ongericimab 150 mg Q2W | Recaticimab 150 mg Q4W | 0.000 | 0.000 | 0.000 | 0.000 | 0.000 | 0.000 | 0.777 | 1.000 | 0.709 | 1.000 |
| Ongericimab 300 mg Q4W | Recaticimab 150 mg Q4W | 0.325 | 1.000 | 0.295 | 1.000 | 0.197 | 1.000 | - | - | - | - |
| Placebo | Recaticimab 150 mg Q4W | 0.000 | 0.000 | 0.000 | 0.000 | 0.000 | 0.000 | 0.868 | 1.000 | 0.797 | 1.000 |
| Ebronucimab 150 mg Q2W | Recaticimab 300 mg Q8W | 0.283 | 1.000 | 0.154 | 1.000 | 0.072 | 1.000 | 0.370 | 1.000 | 0.204 | 1.000 |
| Ebronucimab 450 mg Q4W | Recaticimab 300 mg Q8W | 0.404 | 1.000 | 0.245 | 1.000 | 0.498 | 1.000 | 0.698 | 1.000 | 0.951 | 1.000 |
| Ongericimab 150 mg Q2W | Recaticimab 300 mg Q8W | 0.000 | 0.000 | 0.000 | 0.000 | 0.000 | 0.000 | 0.886 | 1.000 | 0.455 | 1.000 |
| Ongericimab 300 mg Q4W | Recaticimab 300 mg Q8W | 0.293 | 1.000 | 0.211 | 1.000 | 0.025 | 0.769 | - | - | - | - |
| Placebo | Recaticimab 300 mg Q8W | 0.000 | 0.000 | 0.000 | 0.000 | 0.000 | 0.000 | 0.550 | 1.000 | 0.509 | 1.000 |
| Recaticimab 150 mg Q4W | Recaticimab 300 mg Q8W | 0.917 | 1.000 | 0.741 | 1.000 | 0.122 | 1.000 | 0.451 | 1.000 | 0.409 | 1.000 |
| Ebronucimab 150 mg Q2W | Recaticimab 450 mg Q12W | 0.021 | 0.620 | 0.008 | 0.249 | 0.009 | 0.313 | 0.218 | 1.000 | 0.269 | 1.000 |
| Ebronucimab 450 mg Q4W | Recaticimab 450 mg Q12W | 0.039 | 1.000 | 0.017 | 0.454 | 0.133 | 1.000 | 0.471 | 1.000 | 0.885 | 1.000 |
| Ongericimab 150 mg Q2W | Recaticimab 450 mg Q12W | 0.000 | 0.000 | 0.000 | 0.000 | 0.000 | 0.000 | 0.853 | 1.000 | 0.568 | 1.000 |
| Ongericimab 300 mg Q4W | Recaticimab 450 mg Q12W | 0.015 | 0.480 | 0.007 | 0.239 | 0.000 | 0.010 | - | - | - | - |
| Placebo | Recaticimab 450 mg Q12W | 0.000 | 0.000 | 0.000 | 0.000 | 0.000 | 0.000 | 0.995 | 1.000 | 0.832 | 1.000 |
| Recaticimab 150 mg Q4W | Recaticimab 450 mg Q12W | 0.016 | 0.515 | 0.007 | 0.233 | 0.000 | 0.005 | 0.865 | 1.000 | 0.669 | 1.000 |
| Recaticimab 300 mg Q8W | Recaticimab 450 mg Q12W | 0.020 | 0.591 | 0.016 | 0.454 | 0.019 | 0.629 | 0.560 | 1.000 | 0.687 | 1.000 |
| Ebronucimab 150 mg Q2W | Tafolecimab 150 mg Q2W | 0.748 | 1.000 | 0.500 | 1.000 | 0.689 | 1.000 | 0.782 | 1.000 | 0.493 | 1.000 |
| Ebronucimab 450 mg Q4W | Tafolecimab 150 mg Q2W | 0.909 | 1.000 | 0.377 | 1.000 | 0.554 | 1.000 | 0.869 | 1.000 | 0.803 | 1.000 |
| Ongericimab 150 mg Q2W | Tafolecimab 150 mg Q2W | 0.002 | 0.076 | 0.073 | 1.000 | 0.061 | 1.000 | 0.531 | 1.000 | 0.790 | 1.000 |
| Ongericimab 300 mg Q4W | Tafolecimab 150 mg Q2W | 0.828 | 1.000 | 0.307 | 1.000 | 0.963 | 1.000 | - | - | - | - |
| Placebo | Tafolecimab 150 mg Q2W | 0.000 | 0.000 | 0.000 | 0.000 | 0.000 | 0.000 | 0.238 | 1.000 | 0.914 | 1.000 |
| Recaticimab 150 mg Q4W | Tafolecimab 150 mg Q2W | 0.497 | 1.000 | 0.052 | 1.000 | 0.275 | 1.000 | 0.297 | 1.000 | 0.997 | 1.000 |
| Recaticimab 300 mg Q8W | Tafolecimab 150 mg Q2W | 0.458 | 1.000 | 0.035 | 0.806 | 0.063 | 1.000 | 0.551 | 1.000 | 0.743 | 1.000 |
| Recaticimab 450 mg Q12W | Tafolecimab 150 mg Q2W | 0.041 | 1.000 | 0.001 | 0.047 | 0.003 | 0.104 | 0.347 | 1.000 | 0.860 | 1.000 |
| Ebronucimab 150 mg Q2W | Tafolecimab 450 mg Q4W | 0.524 | 1.000 | 0.033 | 0.799 | 0.719 | 1.000 | 0.054 | 1.000 | 0.500 | 1.000 |
| Ebronucimab 450 mg Q4W | Tafolecimab 450 mg Q4W | 0.372 | 1.000 | 0.016 | 0.453 | 0.452 | 1.000 | 0.175 | 1.000 | 0.453 | 1.000 |
| Ongericimab 150 mg Q2W | Tafolecimab 450 mg Q4W | 0.005 | 0.182 | 0.283 | 1.000 | 0.013 | 0.452 | 0.449 | 1.000 | 0.927 | 1.000 |
| Ongericimab 300 mg Q4W | Tafolecimab 450 mg Q4W | 0.371 | 1.000 | 0.004 | 0.136 | 0.837 | 1.000 | - | - | - | - |
| Placebo | Tafolecimab 450 mg Q4W | 0.000 | 0.000 | 0.000 | 0.000 | 0.000 | 0.000 | 0.212 | 1.000 | 0.254 | 1.000 |
| Recaticimab 150 mg Q4W | Tafolecimab 450 mg Q4W | 0.017 | 0.523 | 0.000 | 0.000 | 0.085 | 1.000 | 0.553 | 1.000 | 0.603 | 1.000 |
| Recaticimab 300 mg Q8W | Tafolecimab 450 mg Q4W | 0.013 | 0.436 | 0.000 | 0.000 | 0.005 | 0.174 | 0.222 | 1.000 | 0.210 | 1.000 |
| Recaticimab 450 mg Q12W | Tafolecimab 450 mg Q4W | 0.000 | 0.001 | 0.000 | 0.000 | 0.000 | 0.000 | 0.462 | 1.000 | 0.366 | 1.000 |
| Tafolecimab 150 mg Q2W | Tafolecimab 450 mg Q4W | 0.202 | 1.000 | 0.180 | 1.000 | 0.888 | 1.000 | 0.070 | 1.000 | 0.782 | 1.000 |
| Ebronucimab 150 mg Q2W | Tafolecimab 600 mg Q6W | 0.184 | 1.000 | 0.649 | 1.000 | 0.764 | 1.000 | 0.130 | 1.000 | 0.285 | 1.000 |
| Ebronucimab 450 mg Q4W | Tafolecimab 600 mg Q6W | 0.263 | 1.000 | 0.487 | 1.000 | 0.463 | 1.000 | 0.324 | 1.000 | 0.823 | 1.000 |
| Ongericimab 150 mg Q2W | Tafolecimab 600 mg Q6W | 0.000 | 0.000 | 0.009 | 0.274 | 0.058 | 1.000 | 0.667 | 1.000 | 0.601 | 1.000 |
| Ongericimab 300 mg Q4W | Tafolecimab 600 mg Q6W | 0.188 | 1.000 | 0.396 | 1.000 | 0.823 | 1.000 | - | - | - | - |
| Placebo | Tafolecimab 600 mg Q6W | 0.000 | 0.000 | 0.000 | 0.000 | 0.000 | 0.000 | 0.657 | 1.000 | 0.940 | 1.000 |
| Recaticimab 150 mg Q4W | Tafolecimab 600 mg Q6W | 0.583 | 1.000 | 0.046 | 1.000 | 0.157 | 1.000 | 0.859 | 1.000 | 0.805 | 1.000 |
| Recaticimab 300 mg Q8W | Tafolecimab 600 mg Q6W | 0.624 | 1.000 | 0.028 | 0.734 | 0.023 | 0.731 | 0.460 | 1.000 | 0.658 | 1.000 |
| Recaticimab 450 mg Q12W | Tafolecimab 600 mg Q6W | 0.391 | 1.000 | 0.000 | 0.014 | 0.000 | 0.015 | 0.760 | 1.000 | 0.911 | 1.000 |
| Tafolecimab 150 mg Q2W | Tafolecimab 600 mg Q6W | 0.269 | 1.000 | 0.752 | 1.000 | 0.873 | 1.000 | 0.202 | 1.000 | 0.897 | 1.000 |
| Tafolecimab 450 mg Q4W | Tafolecimab 600 mg Q6W | 0.004 | 0.133 | 0.031 | 0.765 | 0.944 | 1.000 | 0.595 | 1.000 | 0.264 | 1.000 |
| Abbreviations: Q2, 4, 6, 8, 12W, every 2, 4, 6, 8, 12 weeks. | | | | | | | | | | | |

| **Table S8.** The certainty of evidence in LDL-C outcome. | | | | | | | | |
| --- | --- | --- | --- | --- | --- | --- | --- | --- |
| Comparison | Number of studies | Within-study bias | Reporting bias | Indirectness | Imprecision | Heterogeneity | Incoherence | Confidence rating |
| Ebronucimab 150 mg Q2W:Ebronucimab 450 mg Q4W | 1 | No concerns | Low risk | No concerns | Some concerns | Some concerns | No concerns | Low |
| Ebronucimab 150 mg Q2W:Placebo | 1 | No concerns | Low risk | No concerns | No concerns | No concerns | No concerns | High |
| Ebronucimab 450 mg Q4W:Placebo | 1 | No concerns | Low risk | No concerns | No concerns | No concerns | No concerns | High |
| Ongericimab 150 mg Q2W:Ongericimab 300 mg Q4W | 1 | Major concerns | Low risk | No concerns | No concerns | No concerns | No concerns | Low |
| Ongericimab 150 mg Q2W:Placebo | 2 | Major concerns | Low risk | No concerns | No concerns | No concerns | No concerns | Low |
| Ongericimab 300 mg Q4W:Placebo | 1 | Major concerns | Low risk | No concerns | No concerns | No concerns | No concerns | Low |
| Placebo:Recaticimab 150 mg Q4W | 2 | Major concerns | Low risk | No concerns | No concerns | No concerns | No concerns | Low |
| Placebo:Recaticimab 300 mg Q8W | 2 | Major concerns | Low risk | No concerns | No concerns | No concerns | No concerns | Low |
| Placebo:Recaticimab 450 mg Q12W | 2 | Major concerns | Low risk | No concerns | No concerns | No concerns | No concerns | Low |
| Placebo:Tafolecimab 150 mg Q2W | 1 | Some concerns | Low risk | No concerns | No concerns | No concerns | No concerns | Moderate |
| Placebo:Tafolecimab 450 mg Q4W | 3 | Some concerns | Low risk | No concerns | No concerns | No concerns | No concerns | Moderate |
| Placebo:Tafolecimab 600 mg Q6W | 1 | Some concerns | Low risk | No concerns | No concerns | No concerns | No concerns | Moderate |
| Recaticimab 150 mg Q4W:Recaticimab 300 mg Q8W | 2 | Major concerns | Low risk | No concerns | No concerns | Major concerns | No concerns | Very low |
| Recaticimab 150 mg Q4W:Recaticimab 450 mg Q12W | 2 | Major concerns | Low risk | No concerns | No concerns | Some concerns | No concerns | Very low |
| Recaticimab 300 mg Q8W:Recaticimab 450 mg Q12W | 2 | Major concerns | Low risk | No concerns | No concerns | Some concerns | No concerns | Very low |
| Tafolecimab 150 mg Q2W:Tafolecimab 450 mg Q4W | 1 | Some concerns | Low risk | No concerns | Some concerns | No concerns | No concerns | Low |
| Tafolecimab 450 mg Q4W:Tafolecimab 600 mg Q6W | 1 | Some concerns | Low risk | No concerns | No concerns | Some concerns | No concerns | Low |
| Ebronucimab 150 mg Q2W:Ongericimab 150 mg Q2W | 0 | Some concerns | Low risk | No concerns | No concerns | Some concerns | No concerns | Low |
| Ebronucimab 150 mg Q2W:Ongericimab 300 mg Q4W | 0 | Some concerns | Low risk | No concerns | Major concerns | No concerns | No concerns | Very low |
| Ebronucimab 150 mg Q2W:Recaticimab 150 mg Q4W | 0 | Some concerns | Low risk | No concerns | Some concerns | Some concerns | No concerns | Very low |
| Ebronucimab 150 mg Q2W:Recaticimab 300 mg Q8W | 0 | Some concerns | Low risk | No concerns | Some concerns | Some concerns | No concerns | Very low |
| Ebronucimab 150 mg Q2W:Recaticimab 450 mg Q12W | 0 | Some concerns | Low risk | No concerns | No concerns | Some concerns | No concerns | Low |
| Ebronucimab 150 mg Q2W:Tafolecimab 150 mg Q2W | 0 | Some concerns | Low risk | No concerns | Major concerns | No concerns | No concerns | Very low |
| Ebronucimab 150 mg Q2W:Tafolecimab 450 mg Q4W | 0 | No concerns | Low risk | No concerns | Some concerns | Some concerns | No concerns | Low |
| Ebronucimab 150 mg Q2W:Tafolecimab 600 mg Q6W | 0 | Some concerns | Low risk | No concerns | Some concerns | No concerns | No concerns | Low |
| Ebronucimab 450 mg Q4W:Ongericimab 150 mg Q2W | 0 | Some concerns | Low risk | No concerns | No concerns | Some concerns | No concerns | Low |
| Ebronucimab 450 mg Q4W:Ongericimab 300 mg Q4W | 0 | Some concerns | Low risk | No concerns | Major concerns | No concerns | No concerns | Very low |
| Ebronucimab 450 mg Q4W:Recaticimab 150 mg Q4W | 0 | Some concerns | Low risk | No concerns | Some concerns | Some concerns | No concerns | Very low |
| Ebronucimab 450 mg Q4W:Recaticimab 300 mg Q8W | 0 | Some concerns | Low risk | No concerns | Some concerns | Some concerns | No concerns | Very low |
| Ebronucimab 450 mg Q4W:Recaticimab 450 mg Q12W | 0 | Some concerns | Low risk | No concerns | No concerns | Some concerns | No concerns | Low |
| Ebronucimab 450 mg Q4W:Tafolecimab 150 mg Q2W | 0 | Some concerns | Low risk | No concerns | Major concerns | No concerns | No concerns | Very low |
| Ebronucimab 450 mg Q4W:Tafolecimab 450 mg Q4W | 0 | No concerns | Low risk | No concerns | Some concerns | Some concerns | No concerns | Low |
| Ebronucimab 450 mg Q4W:Tafolecimab 600 mg Q6W | 0 | Some concerns | Low risk | No concerns | Some concerns | Some concerns | No concerns | Very low |
| Ongericimab 150 mg Q2W:Recaticimab 150 mg Q4W | 0 | Major concerns | Low risk | No concerns | No concerns | No concerns | No concerns | Low |
| Ongericimab 150 mg Q2W:Recaticimab 300 mg Q8W | 0 | Major concerns | Low risk | No concerns | No concerns | No concerns | No concerns | Low |
| Ongericimab 150 mg Q2W:Recaticimab 450 mg Q12W | 0 | Major concerns | Low risk | No concerns | No concerns | No concerns | No concerns | Low |
| Ongericimab 150 mg Q2W:Tafolecimab 150 mg Q2W | 0 | Some concerns | Low risk | No concerns | No concerns | No concerns | No concerns | Moderate |
| Ongericimab 150 mg Q2W:Tafolecimab 450 mg Q4W | 0 | Some concerns | Low risk | No concerns | No concerns | Some concerns | No concerns | Low |
| Ongericimab 150 mg Q2W:Tafolecimab 600 mg Q6W | 0 | Some concerns | Low risk | No concerns | No concerns | No concerns | No concerns | Moderate |
| Ongericimab 300 mg Q4W:Recaticimab 150 mg Q4W | 0 | Major concerns | Low risk | No concerns | Some concerns | No concerns | No concerns | Very low |
| Ongericimab 300 mg Q4W:Recaticimab 300 mg Q8W | 0 | Major concerns | Low risk | No concerns | Some concerns | No concerns | No concerns | Very low |
| Ongericimab 300 mg Q4W:Recaticimab 450 mg Q12W | 0 | Major concerns | Low risk | No concerns | No concerns | Some concerns | No concerns | Very low |
| Ongericimab 300 mg Q4W:Tafolecimab 150 mg Q2W | 0 | Some concerns | Low risk | No concerns | Major concerns | No concerns | No concerns | Very low |
| Ongericimab 300 mg Q4W:Tafolecimab 450 mg Q4W | 0 | Some concerns | Low risk | No concerns | Some concerns | Some concerns | No concerns | Very low |
| Ongericimab 300 mg Q4W:Tafolecimab 600 mg Q6W | 0 | Some concerns | Low risk | No concerns | Some concerns | No concerns | No concerns | Low |
| Recaticimab 150 mg Q4W:Tafolecimab 150 mg Q2W | 0 | Some concerns | Low risk | No concerns | Some concerns | Some concerns | No concerns | Very low |
| Recaticimab 150 mg Q4W:Tafolecimab 450 mg Q4W | 0 | Some concerns | Low risk | No concerns | No concerns | Some concerns | No concerns | Low |
| Recaticimab 150 mg Q4W:Tafolecimab 600 mg Q6W | 0 | Some concerns | Low risk | No concerns | Some concerns | Some concerns | No concerns | Very low |
| Recaticimab 300 mg Q8W:Tafolecimab 150 mg Q2W | 0 | Some concerns | Low risk | No concerns | Some concerns | Some concerns | No concerns | Very low |
| Recaticimab 300 mg Q8W:Tafolecimab 450 mg Q4W | 0 | Some concerns | Low risk | No concerns | No concerns | Some concerns | No concerns | Low |
| Recaticimab 300 mg Q8W:Tafolecimab 600 mg Q6W | 0 | Some concerns | Low risk | No concerns | Some concerns | Some concerns | No concerns | Very low |
| Recaticimab 450 mg Q12W:Tafolecimab 150 mg Q2W | 0 | Some concerns | Low risk | No concerns | No concerns | Some concerns | No concerns | Low |
| Recaticimab 450 mg Q12W:Tafolecimab 450 mg Q4W | 0 | Some concerns | Low risk | No concerns | No concerns | No concerns | No concerns | Moderate |
| Recaticimab 450 mg Q12W:Tafolecimab 600 mg Q6W | 0 | Some concerns | Low risk | No concerns | Some concerns | Some concerns | No concerns | Very low |
| Tafolecimab 150 mg Q2W:Tafolecimab 600 mg Q6W | 0 | Some concerns | Low risk | No concerns | Some concerns | Some concerns | No concerns | Very low |
| Abbreviations: Q2, 4, 6, 8, 12W, every 2, 4, 6, 8, 12 weeks. | | | | | | | | |

| **Table S9.** The certainty of evidence in ApoB outcome. | | | | | | | | |
| --- | --- | --- | --- | --- | --- | --- | --- | --- |
| Comparison | No. of studies | Within-study bias | Reporting bias | Indirectness | Imprecision | Heterogeneity | Incoherence | Confidence rating |
| Ebronucimab 150 mg Q2W:Ebronucimab 450 mg Q4W | 1 | No concerns | Low risk | No concerns | No concerns | Some concerns | No concerns | Moderate |
| Ebronucimab 150 mg Q2W:Placebo | 1 | No concerns | Low risk | No concerns | No concerns | No concerns | No concerns | High |
| Ebronucimab 450 mg Q4W:Placebo | 1 | No concerns | Low risk | No concerns | No concerns | No concerns | No concerns | High |
| Ongericimab 150 mg Q2W:Ongericimab 300 mg Q4W | 1 | Major concerns | Low risk | No concerns | No concerns | No concerns | No concerns | Low |
| Ongericimab 150 mg Q2W:Placebo | 2 | Major concerns | Low risk | No concerns | No concerns | No concerns | No concerns | Low |
| Ongericimab 300 mg Q4W:Placebo | 1 | Major concerns | Low risk | No concerns | No concerns | No concerns | No concerns | Low |
| Placebo:Recaticimab 150 mg Q4W | 2 | Major concerns | Low risk | No concerns | No concerns | No concerns | No concerns | Low |
| Placebo:Recaticimab 300 mg Q8W | 2 | Major concerns | Low risk | No concerns | No concerns | No concerns | No concerns | Low |
| Placebo:Recaticimab 450 mg Q12W | 2 | Major concerns | Low risk | No concerns | No concerns | No concerns | No concerns | Low |
| Placebo:Tafolecimab 150 mg Q2W | 1 | Some concerns | Low risk | No concerns | No concerns | No concerns | No concerns | Moderate |
| Placebo:Tafolecimab 450 mg Q4W | 3 | Some concerns | Low risk | No concerns | No concerns | No concerns | No concerns | Moderate |
| Placebo:Tafolecimab 600 mg Q6W | 1 | Some concerns | Low risk | No concerns | No concerns | No concerns | No concerns | Moderate |
| Recaticimab 150 mg Q4W:Recaticimab 300 mg Q8W | 2 | Major concerns | Low risk | No concerns | No concerns | No concerns | No concerns | Low |
| Recaticimab 150 mg Q4W:Recaticimab 450 mg Q12W | 2 | Major concerns | Low risk | No concerns | No concerns | Some concerns | No concerns | Very low |
| Recaticimab 300 mg Q8W:Recaticimab 450 mg Q12W | 2 | Major concerns | Low risk | No concerns | No concerns | Some concerns | No concerns | Very low |
| Tafolecimab 150 mg Q2W:Tafolecimab 450 mg Q4W | 1 | Some concerns | Low risk | No concerns | Some concerns | No concerns | No concerns | Low |
| Tafolecimab 450 mg Q4W:Tafolecimab 600 mg Q6W | 1 | Some concerns | Low risk | No concerns | No concerns | Some concerns | No concerns | Low |
| Ebronucimab 150 mg Q2W:Ongericimab 150 mg Q2W | 0 | Some concerns | Low risk | No concerns | No concerns | Some concerns | No concerns | Low |
| Ebronucimab 150 mg Q2W:Ongericimab 300 mg Q4W | 0 | Some concerns | Low risk | No concerns | Some concerns | Some concerns | No concerns | Very low |
| Ebronucimab 150 mg Q2W:Recaticimab 150 mg Q4W | 0 | Some concerns | Low risk | No concerns | Some concerns | No concerns | No concerns | Low |
| Ebronucimab 150 mg Q2W:Recaticimab 300 mg Q8W | 0 | Some concerns | Low risk | No concerns | Some concerns | No concerns | No concerns | Low |
| Ebronucimab 150 mg Q2W:Recaticimab 450 mg Q12W | 0 | Some concerns | Low risk | No concerns | No concerns | Some concerns | No concerns | Low |
| Ebronucimab 150 mg Q2W:Tafolecimab 150 mg Q2W | 0 | Some concerns | Low risk | No concerns | Some concerns | Some concerns | No concerns | Very low |
| Ebronucimab 150 mg Q2W:Tafolecimab 450 mg Q4W | 0 | No concerns | Low risk | No concerns | No concerns | Some concerns | No concerns | Moderate |
| Ebronucimab 150 mg Q2W:Tafolecimab 600 mg Q6W | 0 | Some concerns | Low risk | No concerns | Some concerns | Some concerns | No concerns | Very low |
| Ebronucimab 450 mg Q4W:Ongericimab 150 mg Q2W | 0 | Some concerns | Low risk | No concerns | No concerns | No concerns | No concerns | Moderate |
| Ebronucimab 450 mg Q4W:Ongericimab 300 mg Q4W | 0 | Some concerns | Low risk | No concerns | No concerns | Major concerns | No concerns | Very low |
| Ebronucimab 450 mg Q4W:Recaticimab 150 mg Q4W | 0 | Some concerns | Low risk | No concerns | Some concerns | No concerns | No concerns | Low |
| Ebronucimab 450 mg Q4W:Recaticimab 300 mg Q8W | 0 | Some concerns | Low risk | No concerns | Some concerns | No concerns | No concerns | Low |
| Ebronucimab 450 mg Q4W:Recaticimab 450 mg Q12W | 0 | Some concerns | Low risk | No concerns | No concerns | Some concerns | No concerns | Low |
| Ebronucimab 450 mg Q4W:Tafolecimab 150 mg Q2W | 0 | Some concerns | Low risk | No concerns | Some concerns | Some concerns | No concerns | Very low |
| Ebronucimab 450 mg Q4W:Tafolecimab 450 mg Q4W | 0 | No concerns | Low risk | No concerns | No concerns | Some concerns | No concerns | Moderate |
| Ebronucimab 450 mg Q4W:Tafolecimab 600 mg Q6W | 0 | Some concerns | Low risk | No concerns | Some concerns | Some concerns | No concerns | Very low |
| Ongericimab 150 mg Q2W:Recaticimab 150 mg Q4W | 0 | Major concerns | Low risk | No concerns | No concerns | No concerns | No concerns | Low |
| Ongericimab 150 mg Q2W:Recaticimab 300 mg Q8W | 0 | Major concerns | Low risk | No concerns | No concerns | No concerns | No concerns | Low |
| Ongericimab 150 mg Q2W:Recaticimab 450 mg Q12W | 0 | Major concerns | Low risk | No concerns | No concerns | No concerns | No concerns | Low |
| Ongericimab 150 mg Q2W:Tafolecimab 150 mg Q2W | 0 | Some concerns | Low risk | No concerns | Some concerns | No concerns | No concerns | Low |
| Ongericimab 150 mg Q2W:Tafolecimab 450 mg Q4W | 0 | Some concerns | Low risk | No concerns | No concerns | Some concerns | No concerns | Low |
| Ongericimab 150 mg Q2W:Tafolecimab 600 mg Q6W | 0 | Some concerns | Low risk | No concerns | No concerns | Some concerns | No concerns | Low |
| Ongericimab 300 mg Q4W:Recaticimab 150 mg Q4W | 0 | Major concerns | Low risk | No concerns | Some concerns | No concerns | No concerns | Very low |
| Ongericimab 300 mg Q4W:Recaticimab 300 mg Q8W | 0 | Major concerns | Low risk | No concerns | Some concerns | No concerns | No concerns | Very low |
| Ongericimab 300 mg Q4W:Recaticimab 450 mg Q12W | 0 | Major concerns | Low risk | No concerns | No concerns | Some concerns | No concerns | Very low |
| Ongericimab 300 mg Q4W:Tafolecimab 150 mg Q2W | 0 | Some concerns | Low risk | No concerns | Some concerns | No concerns | No concerns | Low |
| Ongericimab 300 mg Q4W:Tafolecimab 450 mg Q4W | 0 | Some concerns | Low risk | No concerns | No concerns | Some concerns | No concerns | Low |
| Ongericimab 300 mg Q4W:Tafolecimab 600 mg Q6W | 0 | Some concerns | Low risk | No concerns | Some concerns | No concerns | No concerns | Low |
| Recaticimab 150 mg Q4W:Tafolecimab 150 mg Q2W | 0 | Some concerns | Low risk | No concerns | Some concerns | No concerns | No concerns | Low |
| Recaticimab 150 mg Q4W:Tafolecimab 450 mg Q4W | 0 | Some concerns | Low risk | No concerns | No concerns | No concerns | No concerns | Moderate |
| Recaticimab 150 mg Q4W:Tafolecimab 600 mg Q6W | 0 | Some concerns | Low risk | No concerns | No concerns | Some concerns | No concerns | Low |
| Recaticimab 300 mg Q8W:Tafolecimab 150 mg Q2W | 0 | Some concerns | Low risk | No concerns | No concerns | Some concerns | No concerns | Low |
| Recaticimab 300 mg Q8W:Tafolecimab 450 mg Q4W | 0 | Some concerns | Low risk | No concerns | No concerns | No concerns | No concerns | Moderate |
| Recaticimab 300 mg Q8W:Tafolecimab 600 mg Q6W | 0 | Some concerns | Low risk | No concerns | No concerns | Some concerns | No concerns | Low |
| Recaticimab 450 mg Q12W:Tafolecimab 150 mg Q2W | 0 | Some concerns | Low risk | No concerns | No concerns | No concerns | No concerns | Moderate |
| Recaticimab 450 mg Q12W:Tafolecimab 450 mg Q4W | 0 | Some concerns | Low risk | No concerns | No concerns | No concerns | No concerns | Moderate |
| Recaticimab 450 mg Q12W:Tafolecimab 600 mg Q6W | 0 | Some concerns | Low risk | No concerns | No concerns | No concerns | No concerns | Moderate |
| Tafolecimab 150 mg Q2W:Tafolecimab 600 mg Q6W | 0 | Some concerns | Low risk | No concerns | Some concerns | Some concerns | No concerns | Very low |
| Abbreviations: Q2, 4, 6, 8, 12W, every 2, 4, 6, 8, 12 weeks. | | | | | | | | |

| **Table S10.** The certainty of evidence in Lp(a) outcome. | | | | | | | | |
| --- | --- | --- | --- | --- | --- | --- | --- | --- |
| Comparison | No. of studies | Within-study bias | Reporting bias | Indirectness | Imprecision | Heterogeneity | Incoherence | Confidence rating |
| Ebronucimab 150 mg Q2W:Ebronucimab 450 mg Q4W | 1 | No concerns | Low risk | No concerns | Major concerns | No concerns | No concerns | Low |
| Ebronucimab 150 mg Q2W:Placebo | 1 | No concerns | Low risk | No concerns | No concerns | No concerns | No concerns | High |
| Ebronucimab 450 mg Q4W:Placebo | 1 | No concerns | Low risk | No concerns | No concerns | No concerns | No concerns | High |
| Ongericimab 150 mg Q2W:Ongericimab 300 mg Q4W | 1 | Major concerns | Low risk | No concerns | No concerns | Some concerns | No concerns | Very low |
| Ongericimab 150 mg Q2W:Placebo | 2 | Major concerns | Low risk | No concerns | No concerns | No concerns | No concerns | Low |
| Ongericimab 300 mg Q4W:Placebo | 1 | Major concerns | Low risk | No concerns | No concerns | No concerns | No concerns | Low |
| Placebo:Recaticimab 150 mg Q4W | 2 | Some concerns | Low risk | No concerns | No concerns | No concerns | No concerns | Moderate |
| Placebo:Recaticimab 300 mg Q8W | 2 | Some concerns | Low risk | No concerns | No concerns | No concerns | No concerns | Moderate |
| Placebo:Recaticimab 450 mg Q12W | 2 | Some concerns | Low risk | No concerns | No concerns | No concerns | No concerns | Moderate |
| Placebo:Tafolecimab 150 mg Q2W | 1 | Some concerns | Low risk | No concerns | No concerns | No concerns | No concerns | Moderate |
| Placebo:Tafolecimab 450 mg Q4W | 3 | Some concerns | Low risk | No concerns | No concerns | No concerns | No concerns | Moderate |
| Placebo:Tafolecimab 600 mg Q6W | 1 | Some concerns | Low risk | No concerns | No concerns | No concerns | Some concerns | Low |
| Recaticimab 150 mg Q4W:Recaticimab 300 mg Q8W | 2 | Some concerns | Low risk | No concerns | Some concerns | No concerns | No concerns | Low |
| Recaticimab 150 mg Q4W:Recaticimab 450 mg Q12W | 2 | Some concerns | Low risk | No concerns | No concerns | No concerns | No concerns | Moderate |
| Recaticimab 300 mg Q8W:Recaticimab 450 mg Q12W | 2 | Some concerns | Low risk | No concerns | No concerns | Some concerns | No concerns | Low |
| Tafolecimab 150 mg Q2W:Tafolecimab 450 mg Q4W | 1 | Some concerns | Low risk | No concerns | Major concerns | No concerns | No concerns | Very low |
| Tafolecimab 450 mg Q4W:Tafolecimab 600 mg Q6W | 1 | Some concerns | Low risk | No concerns | Major concerns | No concerns | Major concerns | Very low |
| Ebronucimab 150 mg Q2W:Ongericimab 150 mg Q2W | 0 | Some concerns | Low risk | No concerns | Major concerns | No concerns | No concerns | Very low |
| Ebronucimab 150 mg Q2W:Ongericimab 300 mg Q4W | 0 | Some concerns | Low risk | No concerns | Major concerns | No concerns | No concerns | Very low |
| Ebronucimab 150 mg Q2W:Recaticimab 150 mg Q4W | 0 | Some concerns | Low risk | No concerns | Some concerns | Some concerns | No concerns | Very low |
| Ebronucimab 150 mg Q2W:Recaticimab 300 mg Q8W | 0 | Some concerns | Low risk | No concerns | Some concerns | Some concerns | No concerns | Very low |
| Ebronucimab 150 mg Q2W:Recaticimab 450 mg Q12W | 0 | Some concerns | Low risk | No concerns | No concerns | Some concerns | No concerns | Low |
| Ebronucimab 150 mg Q2W:Tafolecimab 150 mg Q2W | 0 | Some concerns | Low risk | No concerns | Major concerns | No concerns | No concerns | Very low |
| Ebronucimab 150 mg Q2W:Tafolecimab 450 mg Q4W | 0 | No concerns | Low risk | No concerns | Major concerns | No concerns | No concerns | Low |
| Ebronucimab 150 mg Q2W:Tafolecimab 600 mg Q6W | 0 | Some concerns | Low risk | No concerns | Major concerns | No concerns | No concerns | Very low |
| Ebronucimab 450 mg Q4W:Ongericimab 150 mg Q2W | 0 | Some concerns | Low risk | No concerns | No concerns | Some concerns | No concerns | Low |
| Ebronucimab 450 mg Q4W:Ongericimab 300 mg Q4W | 0 | Some concerns | Low risk | No concerns | Major concerns | No concerns | No concerns | Very low |
| Ebronucimab 450 mg Q4W:Recaticimab 150 mg Q4W | 0 | Some concerns | Low risk | No concerns | Major concerns | No concerns | No concerns | Very low |
| Ebronucimab 450 mg Q4W:Recaticimab 300 mg Q8W | 0 | Some concerns | Low risk | No concerns | Major concerns | No concerns | No concerns | Very low |
| Ebronucimab 450 mg Q4W:Recaticimab 450 mg Q12W | 0 | Some concerns | Low risk | No concerns | Some concerns | Some concerns | No concerns | Very low |
| Ebronucimab 450 mg Q4W:Tafolecimab 150 mg Q2W | 0 | Some concerns | Low risk | No concerns | Major concerns | No concerns | No concerns | Very low |
| Ebronucimab 450 mg Q4W:Tafolecimab 450 mg Q4W | 0 | No concerns | Low risk | No concerns | Major concerns | No concerns | No concerns | Low |
| Ebronucimab 450 mg Q4W:Tafolecimab 600 mg Q6W | 0 | Some concerns | Low risk | No concerns | Major concerns | No concerns | No concerns | Very low |
| Ongericimab 150 mg Q2W:Recaticimab 150 mg Q4W | 0 | Major concerns | Low risk | No concerns | No concerns | No concerns | No concerns | Low |
| Ongericimab 150 mg Q2W:Recaticimab 300 mg Q8W | 0 | Major concerns | Low risk | No concerns | No concerns | No concerns | No concerns | Low |
| Ongericimab 150 mg Q2W:Recaticimab 450 mg Q12W | 0 | Major concerns | Low risk | No concerns | No concerns | No concerns | No concerns | Low |
| Ongericimab 150 mg Q2W:Tafolecimab 150 mg Q2W | 0 | Some concerns | Low risk | No concerns | Some concerns | No concerns | No concerns | Low |
| Ongericimab 150 mg Q2W:Tafolecimab 450 mg Q4W | 0 | Some concerns | Low risk | No concerns | No concerns | Some concerns | No concerns | Low |
| Ongericimab 150 mg Q2W:Tafolecimab 600 mg Q6W | 0 | Some concerns | Low risk | No concerns | Some concerns | No concerns | No concerns | Low |
| Ongericimab 300 mg Q4W:Recaticimab 150 mg Q4W | 0 | Major concerns | Low risk | No concerns | Some concerns | Some concerns | No concerns | Very low |
| Ongericimab 300 mg Q4W:Recaticimab 300 mg Q8W | 0 | Major concerns | Low risk | No concerns | No concerns | Some concerns | No concerns | Very low |
| Ongericimab 300 mg Q4W:Recaticimab 450 mg Q12W | 0 | Major concerns | Low risk | No concerns | No concerns | No concerns | No concerns | Low |
| Ongericimab 300 mg Q4W:Tafolecimab 150 mg Q2W | 0 | Some concerns | Low risk | No concerns | Major concerns | No concerns | No concerns | Very low |
| Ongericimab 300 mg Q4W:Tafolecimab 450 mg Q4W | 0 | Some concerns | Low risk | No concerns | Major concerns | No concerns | No concerns | Very low |
| Ongericimab 300 mg Q4W:Tafolecimab 600 mg Q6W | 0 | Some concerns | Low risk | No concerns | Major concerns | No concerns | No concerns | Very low |
| Recaticimab 150 mg Q4W:Tafolecimab 150 mg Q2W | 0 | Some concerns | Low risk | No concerns | Some concerns | Some concerns | No concerns | Very low |
| Recaticimab 150 mg Q4W:Tafolecimab 450 mg Q4W | 0 | Some concerns | Low risk | No concerns | Some concerns | No concerns | No concerns | Low |
| Recaticimab 150 mg Q4W:Tafolecimab 600 mg Q6W | 0 | Some concerns | Low risk | No concerns | Some concerns | Some concerns | No concerns | Very low |
| Recaticimab 300 mg Q8W:Tafolecimab 150 mg Q2W | 0 | Some concerns | Low risk | No concerns | Some concerns | No concerns | No concerns | Low |
| Recaticimab 300 mg Q8W:Tafolecimab 450 mg Q4W | 0 | Some concerns | Low risk | No concerns | No concerns | Some concerns | No concerns | Low |
| Recaticimab 300 mg Q8W:Tafolecimab 600 mg Q6W | 0 | Some concerns | Low risk | No concerns | No concerns | Some concerns | No concerns | Low |
| Recaticimab 450 mg Q12W:Tafolecimab 150 mg Q2W | 0 | Some concerns | Low risk | No concerns | No concerns | No concerns | No concerns | Moderate |
| Recaticimab 450 mg Q12W:Tafolecimab 450 mg Q4W | 0 | Some concerns | Low risk | No concerns | No concerns | No concerns | No concerns | Moderate |
| Recaticimab 450 mg Q12W:Tafolecimab 600 mg Q6W | 0 | Some concerns | Low risk | No concerns | No concerns | No concerns | No concerns | Moderate |
| Tafolecimab 150 mg Q2W:Tafolecimab 600 mg Q6W | 0 | Some concerns | Low risk | No concerns | Major concerns | No concerns | No concerns | Very low |
| Abbreviations: Q2, 4, 6, 8, 12W, every 2, 4, 6, 8, 12 weeks. | | | | | | | | |

| **Table S11.** The certainty of evidence in TEAEs outcome. | | | | | | | | |
| --- | --- | --- | --- | --- | --- | --- | --- | --- |
| Comparison | No. of studies | Within-study bias | Reporting bias | Indirectness | Imprecision | Heterogeneity | Incoherence | Confidence rating |
| Ebronucimab 150 mg Q2W:Ebronucimab 450 mg Q4W | 1 | No concerns | Low risk | No concerns | Some concerns | Some concerns | No concerns | Low |
| Ebronucimab 150 mg Q2W:Placebo | 1 | No concerns | Low risk | No concerns | Some concerns | Some concerns | No concerns | Low |
| Ebronucimab 450 mg Q4W:Placebo | 1 | No concerns | Low risk | No concerns | Some concerns | Some concerns | No concerns | Low |
| Ongericimab 150 mg Q2W:Placebo | 1 | Some concerns | Low risk | No concerns | Major concerns | No concerns | No concerns | Very low |
| Placebo:Recaticimab 150 mg Q4W | 1 | Some concerns | Low risk | No concerns | No concerns | Major concerns | No concerns | Very low |
| Placebo:Recaticimab 300 mg Q8W | 1 | Some concerns | Low risk | No concerns | Some concerns | Some concerns | No concerns | Very low |
| Placebo:Recaticimab 450 mg Q12W | 1 | Some concerns | Low risk | No concerns | No concerns | Major concerns | No concerns | Very low |
| Placebo:Tafolecimab 150 mg Q2W | 1 | Some concerns | Low risk | No concerns | Some concerns | Some concerns | No concerns | Very low |
| Placebo:Tafolecimab 450 mg Q4W | 3 | Some concerns | Low risk | No concerns | Some concerns | Some concerns | No concerns | Very low |
| Placebo:Tafolecimab 600 mg Q6W | 1 | Some concerns | Low risk | No concerns | Some concerns | Some concerns | No concerns | Very low |
| Recaticimab 150 mg Q4W:Recaticimab 300 mg Q8W | 1 | Some concerns | Low risk | No concerns | Some concerns | Some concerns | No concerns | Very low |
| Recaticimab 150 mg Q4W:Recaticimab 450 mg Q12W | 1 | Some concerns | Low risk | No concerns | No concerns | Major concerns | No concerns | Very low |
| Recaticimab 300 mg Q8W:Recaticimab 450 mg Q12W | 1 | Some concerns | Low risk | No concerns | Some concerns | Some concerns | No concerns | Very low |
| Tafolecimab 150 mg Q2W:Tafolecimab 450 mg Q4W | 1 | Some concerns | Low risk | No concerns | Some concerns | Some concerns | No concerns | Very low |
| Tafolecimab 450 mg Q4W:Tafolecimab 600 mg Q6W | 1 | Some concerns | Low risk | No concerns | Some concerns | Some concerns | No concerns | Very low |
| Ebronucimab 150 mg Q2W:Ongericimab 150 mg Q2W | 0 | No concerns | Low risk | No concerns | Some concerns | Some concerns | No concerns | Low |
| Ebronucimab 150 mg Q2W:Recaticimab 150 mg Q4W | 0 | Some concerns | Low risk | No concerns | Some concerns | Some concerns | No concerns | Very low |
| Ebronucimab 150 mg Q2W:Recaticimab 300 mg Q8W | 0 | Some concerns | Low risk | No concerns | Some concerns | Some concerns | No concerns | Very low |
| Ebronucimab 150 mg Q2W:Recaticimab 450 mg Q12W | 0 | Some concerns | Low risk | No concerns | Some concerns | Some concerns | No concerns | Very low |
| Ebronucimab 150 mg Q2W:Tafolecimab 150 mg Q2W | 0 | Some concerns | Low risk | No concerns | Major concerns | No concerns | No concerns | Very low |
| Ebronucimab 150 mg Q2W:Tafolecimab 450 mg Q4W | 0 | No concerns | Low risk | No concerns | Some concerns | Some concerns | No concerns | Low |
| Ebronucimab 150 mg Q2W:Tafolecimab 600 mg Q6W | 0 | Some concerns | Low risk | No concerns | Some concerns | Some concerns | No concerns | Very low |
| Ebronucimab 450 mg Q4W:Ongericimab 150 mg Q2W | 0 | No concerns | Low risk | No concerns | Major concerns | No concerns | No concerns | Low |
| Ebronucimab 450 mg Q4W:Recaticimab 150 mg Q4W | 0 | Some concerns | Low risk | No concerns | Some concerns | Some concerns | No concerns | Very low |
| Ebronucimab 450 mg Q4W:Recaticimab 300 mg Q8W | 0 | Some concerns | Low risk | No concerns | Major concerns | No concerns | No concerns | Very low |
| Ebronucimab 450 mg Q4W:Recaticimab 450 mg Q12W | 0 | Some concerns | Low risk | No concerns | Some concerns | Some concerns | No concerns | Very low |
| Ebronucimab 450 mg Q4W:Tafolecimab 150 mg Q2W | 0 | Some concerns | Low risk | No concerns | Major concerns | No concerns | No concerns | Very low |
| Ebronucimab 450 mg Q4W:Tafolecimab 450 mg Q4W | 0 | No concerns | Low risk | No concerns | Some concerns | Some concerns | No concerns | Low |
| Ebronucimab 450 mg Q4W:Tafolecimab 600 mg Q6W | 0 | Some concerns | Low risk | No concerns | Some concerns | Some concerns | No concerns | Very low |
| Ongericimab 150 mg Q2W:Recaticimab 150 mg Q4W | 0 | Some concerns | Low risk | No concerns | Major concerns | No concerns | No concerns | Very low |
| Ongericimab 150 mg Q2W:Recaticimab 300 mg Q8W | 0 | Some concerns | Low risk | No concerns | Major concerns | No concerns | No concerns | Very low |
| Ongericimab 150 mg Q2W:Recaticimab 450 mg Q12W | 0 | Some concerns | Low risk | No concerns | Major concerns | No concerns | No concerns | Very low |
| Ongericimab 150 mg Q2W:Tafolecimab 150 mg Q2W | 0 | Some concerns | Low risk | No concerns | Major concerns | No concerns | No concerns | Very low |
| Ongericimab 150 mg Q2W:Tafolecimab 450 mg Q4W | 0 | Some concerns | Low risk | No concerns | Some concerns | Some concerns | No concerns | Very low |
| Ongericimab 150 mg Q2W:Tafolecimab 600 mg Q6W | 0 | Some concerns | Low risk | No concerns | Major concerns | No concerns | No concerns | Very low |
| Recaticimab 150 mg Q4W:Tafolecimab 150 mg Q2W | 0 | Some concerns | Low risk | No concerns | Some concerns | Some concerns | No concerns | Very low |
| Recaticimab 150 mg Q4W:Tafolecimab 450 mg Q4W | 0 | Some concerns | Low risk | No concerns | Some concerns | Some concerns | No concerns | Very low |
| Recaticimab 150 mg Q4W:Tafolecimab 600 mg Q6W | 0 | Some concerns | Low risk | No concerns | Major concerns | No concerns | No concerns | Very low |
| Recaticimab 300 mg Q8W:Tafolecimab 150 mg Q2W | 0 | Some concerns | Low risk | No concerns | Major concerns | No concerns | No concerns | Very low |
| Recaticimab 300 mg Q8W:Tafolecimab 450 mg Q4W | 0 | Some concerns | Low risk | No concerns | Some concerns | Some concerns | No concerns | Very low |
| Recaticimab 300 mg Q8W:Tafolecimab 600 mg Q6W | 0 | Some concerns | Low risk | No concerns | Some concerns | Some concerns | No concerns | Very low |
| Recaticimab 450 mg Q12W:Tafolecimab 150 mg Q2W | 0 | Some concerns | Low risk | No concerns | Some concerns | Some concerns | No concerns | Very low |
| Recaticimab 450 mg Q12W:Tafolecimab 450 mg Q4W | 0 | Some concerns | Low risk | No concerns | Some concerns | Some concerns | No concerns | Very low |
| Recaticimab 450 mg Q12W:Tafolecimab 600 mg Q6W | 0 | Some concerns | Low risk | No concerns | Major concerns | No concerns | No concerns | Very low |
| Tafolecimab 150 mg Q2W:Tafolecimab 600 mg Q6W | 0 | Some concerns | Low risk | No concerns | Some concerns | Some concerns | No concerns | Very low |
| Abbreviations: Q2, 4, 6, 8, 12W, every 2, 4, 6, 8, 12 weeks. | | | | | | | | |

| **Table S12.** The certainty of evidence in serious TEAEs outcome. | | | | | | | | |
| --- | --- | --- | --- | --- | --- | --- | --- | --- |
| Comparison | No. of studies | Within-study bias | Reporting bias | Indirectness | Imprecision | Heterogeneity | Incoherence | Confidence rating |
| Ebronucimab 150 mg Q2W:Ebronucimab 450 mg Q4W | 1 | No concerns | Low risk | No concerns | Major concerns | No concerns | No concerns | Low |
| Ebronucimab 150 mg Q2W:Placebo | 1 | No concerns | Low risk | No concerns | Major concerns | No concerns | No concerns | Low |
| Ebronucimab 450 mg Q4W:Placebo | 1 | No concerns | Low risk | No concerns | Major concerns | No concerns | No concerns | Low |
| Ongericimab 150 mg Q2W:Placebo | 1 | Some concerns | Low risk | No concerns | Major concerns | No concerns | No concerns | Very low |
| Placebo:Recaticimab 150 mg Q4W | 1 | Some concerns | Low risk | No concerns | Major concerns | No concerns | No concerns | Very low |
| Placebo:Recaticimab 300 mg Q8W | 1 | Some concerns | Low risk | No concerns | Major concerns | No concerns | No concerns | Very low |
| Placebo:Recaticimab 450 mg Q12W | 1 | Some concerns | Low risk | No concerns | Major concerns | No concerns | No concerns | Very low |
| Placebo:Tafolecimab 150 mg Q2W | 1 | Some concerns | Low risk | No concerns | Major concerns | No concerns | No concerns | Very low |
| Placebo:Tafolecimab 450 mg Q4W | 3 | Some concerns | Low risk | No concerns | Some concerns | Some concerns | No concerns | Very low |
| Placebo:Tafolecimab 600 mg Q6W | 1 | Some concerns | Low risk | No concerns | Major concerns | No concerns | No concerns | Very low |
| Recaticimab 150 mg Q4W:Recaticimab 300 mg Q8W | 1 | Some concerns | Low risk | No concerns | Major concerns | No concerns | No concerns | Very low |
| Recaticimab 150 mg Q4W:Recaticimab 450 mg Q12W | 1 | Some concerns | Low risk | No concerns | Major concerns | No concerns | No concerns | Very low |
| Recaticimab 300 mg Q8W:Recaticimab 450 mg Q12W | 1 | Some concerns | Low risk | No concerns | Major concerns | No concerns | No concerns | Very low |
| Tafolecimab 150 mg Q2W:Tafolecimab 450 mg Q4W | 1 | Some concerns | Low risk | No concerns | Major concerns | No concerns | No concerns | Very low |
| Tafolecimab 450 mg Q4W:Tafolecimab 600 mg Q6W | 1 | Some concerns | Low risk | No concerns | Some concerns | Some concerns | No concerns | Very low |
| Ebronucimab 150 mg Q2W:Ongericimab 150 mg Q2W | 0 | No concerns | Low risk | No concerns | Major concerns | No concerns | No concerns | Low |
| Ebronucimab 150 mg Q2W:Recaticimab 150 mg Q4W | 0 | Some concerns | Low risk | No concerns | Major concerns | No concerns | No concerns | Very low |
| Ebronucimab 150 mg Q2W:Recaticimab 300 mg Q8W | 0 | Some concerns | Low risk | No concerns | Major concerns | No concerns | No concerns | Very low |
| Ebronucimab 150 mg Q2W:Recaticimab 450 mg Q12W | 0 | Some concerns | Low risk | No concerns | Major concerns | No concerns | No concerns | Very low |
| Ebronucimab 150 mg Q2W:Tafolecimab 150 mg Q2W | 0 | Some concerns | Low risk | No concerns | Major concerns | No concerns | No concerns | Very low |
| Ebronucimab 150 mg Q2W:Tafolecimab 450 mg Q4W | 0 | No concerns | Low risk | No concerns | Major concerns | No concerns | No concerns | Low |
| Ebronucimab 150 mg Q2W:Tafolecimab 600 mg Q6W | 0 | No concerns | Low risk | No concerns | Major concerns | No concerns | No concerns | Low |
| Ebronucimab 450 mg Q4W:Ongericimab 150 mg Q2W | 0 | No concerns | Low risk | No concerns | Major concerns | No concerns | No concerns | Low |
| Ebronucimab 450 mg Q4W:Recaticimab 150 mg Q4W | 0 | Some concerns | Low risk | No concerns | Major concerns | No concerns | No concerns | Very low |
| Ebronucimab 450 mg Q4W:Recaticimab 300 mg Q8W | 0 | Some concerns | Low risk | No concerns | Major concerns | No concerns | No concerns | Very low |
| Ebronucimab 450 mg Q4W:Recaticimab 450 mg Q12W | 0 | Some concerns | Low risk | No concerns | Major concerns | No concerns | No concerns | Very low |
| Ebronucimab 450 mg Q4W:Tafolecimab 150 mg Q2W | 0 | Some concerns | Low risk | No concerns | Major concerns | No concerns | No concerns | Very low |
| Ebronucimab 450 mg Q4W:Tafolecimab 450 mg Q4W | 0 | Some concerns | Low risk | No concerns | Major concerns | No concerns | No concerns | Very low |
| Ebronucimab 450 mg Q4W:Tafolecimab 600 mg Q6W | 0 | Some concerns | Low risk | No concerns | Major concerns | No concerns | No concerns | Very low |
| Ongericimab 150 mg Q2W:Recaticimab 150 mg Q4W | 0 | Some concerns | Low risk | No concerns | Major concerns | No concerns | No concerns | Very low |
| Ongericimab 150 mg Q2W:Recaticimab 300 mg Q8W | 0 | Some concerns | Low risk | No concerns | Major concerns | No concerns | No concerns | Very low |
| Ongericimab 150 mg Q2W:Recaticimab 450 mg Q12W | 0 | Some concerns | Low risk | No concerns | Major concerns | No concerns | No concerns | Very low |
| Ongericimab 150 mg Q2W:Tafolecimab 150 mg Q2W | 0 | Some concerns | Low risk | No concerns | Major concerns | No concerns | No concerns | Very low |
| Ongericimab 150 mg Q2W:Tafolecimab 450 mg Q4W | 0 | Some concerns | Low risk | No concerns | Major concerns | No concerns | No concerns | Very low |
| Ongericimab 150 mg Q2W:Tafolecimab 600 mg Q6W | 0 | Some concerns | Low risk | No concerns | Major concerns | No concerns | No concerns | Very low |
| Recaticimab 150 mg Q4W:Tafolecimab 150 mg Q2W | 0 | Some concerns | Low risk | No concerns | Major concerns | No concerns | No concerns | Very low |
| Recaticimab 150 mg Q4W:Tafolecimab 450 mg Q4W | 0 | Some concerns | Low risk | No concerns | Major concerns | No concerns | No concerns | Very low |
| Recaticimab 150 mg Q4W:Tafolecimab 600 mg Q6W | 0 | Some concerns | Low risk | No concerns | Major concerns | No concerns | No concerns | Very low |
| Recaticimab 300 mg Q8W:Tafolecimab 150 mg Q2W | 0 | Some concerns | Low risk | No concerns | Major concerns | No concerns | No concerns | Very low |
| Recaticimab 300 mg Q8W:Tafolecimab 450 mg Q4W | 0 | Some concerns | Low risk | No concerns | Major concerns | No concerns | No concerns | Very low |
| Recaticimab 300 mg Q8W:Tafolecimab 600 mg Q6W | 0 | Some concerns | Low risk | No concerns | Major concerns | No concerns | No concerns | Very low |
| Recaticimab 450 mg Q12W:Tafolecimab 150 mg Q2W | 0 | Some concerns | Low risk | No concerns | Major concerns | No concerns | No concerns | Very low |
| Recaticimab 450 mg Q12W:Tafolecimab 450 mg Q4W | 0 | Some concerns | Low risk | No concerns | Major concerns | No concerns | No concerns | Very low |
| Recaticimab 450 mg Q12W:Tafolecimab 600 mg Q6W | 0 | Some concerns | Low risk | No concerns | Major concerns | No concerns | No concerns | Very low |
| Tafolecimab 150 mg Q2W:Tafolecimab 600 mg Q6W | 0 | Some concerns | Low risk | No concerns | Major concerns | No concerns | No concerns | Very low |
| Abbreviations: Q2, 4, 6, 8, 12W, every 2, 4, 6, 8, 12 weeks. | | | | | | | | |

**Figure S1.** The forest plot of direct and indirect evidence for estimating the percentage change in LDL-C, %. CI, confidence interval; MD, mean difference; Q2, 4, 6, 8, 12W, every 2, 4, 6, 8, 12 weeks.

**Figure S2.** The forest plot of direct and indirect evidence for estimating the percentage change in ApoB, %. CI, confidence interval; MD, mean difference; Q2, 4, 6, 12W, every 2, 4, 6, 12 weeks.

**Figure S3.** The forest plot of direct and indirect evidence for estimating the percentage change in Lp(a), %. CI, confidence interval; MD, mean difference; Q2, 4, 6W, every 2, 4, 6 weeks.

**Figure S4.** The forest plot of direct and indirect evidence for estimating the risk ratio of TEAEs. CI, confidence interval; Q2, 4, 6W, every 2, 4, 6 weeks; RR, risk ratio.

**Figure S5.** The forest plot of direct and indirect evidence for estimating the risk ratio of serious TEAEs. CI, confidence interval; Q2, 4, 6W, every 2, 4, 6 weeks; RR, risk ratio.


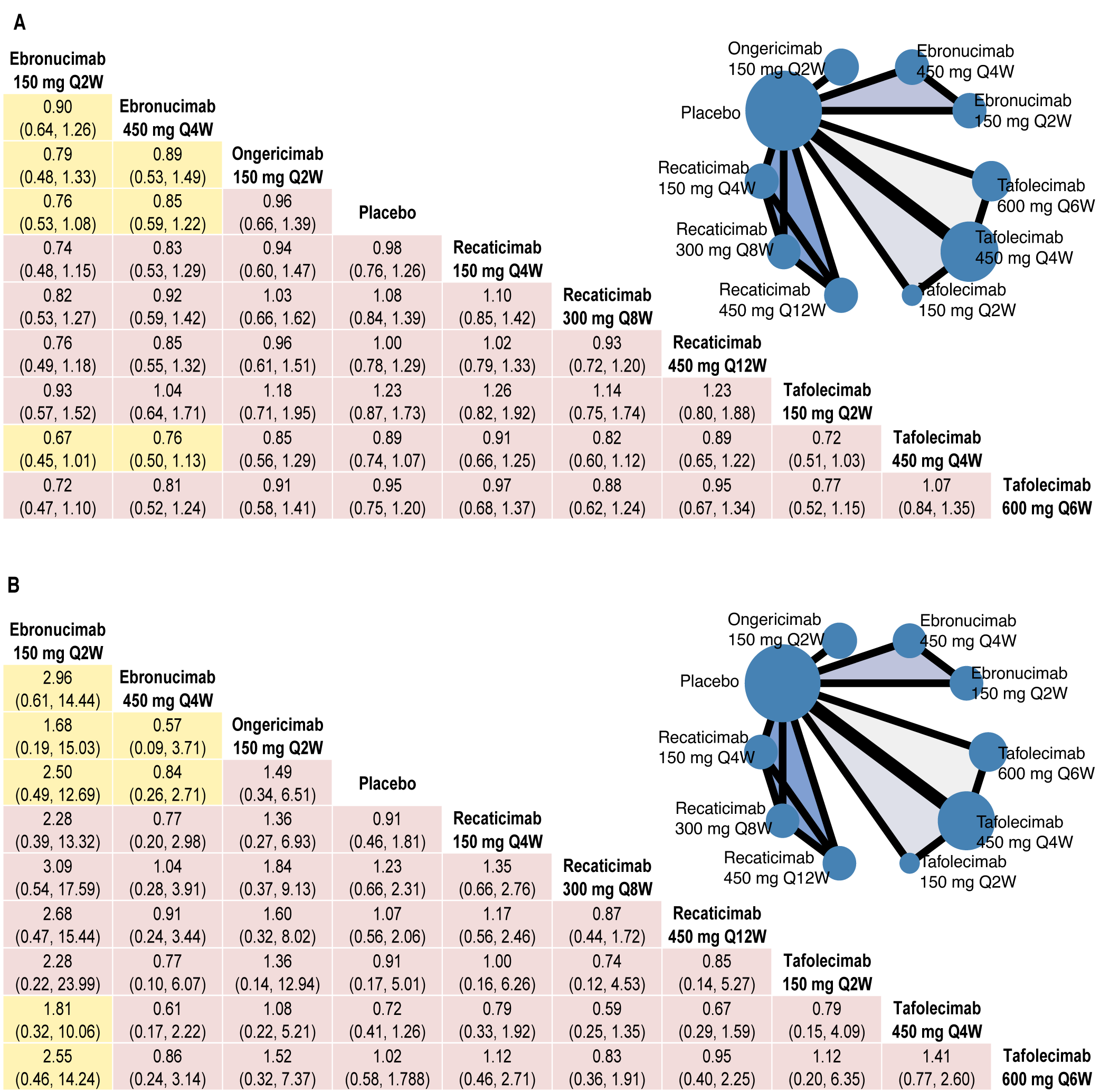


**Figure S6.** Network geometry and league table of **(A)** TEAEs; **(B)** serious TEAEs. The upper right panel shows the corresponding network geometry of each outcome. The lower left panel shows the corresponding league table of each outcome, comparisons should be read from right to left. The results are represented in risk ratios (RRs) and 95% confidence intervals (CIs). Shades in yellow mean “low” confidence rating; shades in red mean “very low” confidence rating. Q2, 4, 6, 8, 12W, every 2, 4, 6, 8, 12 weeks.


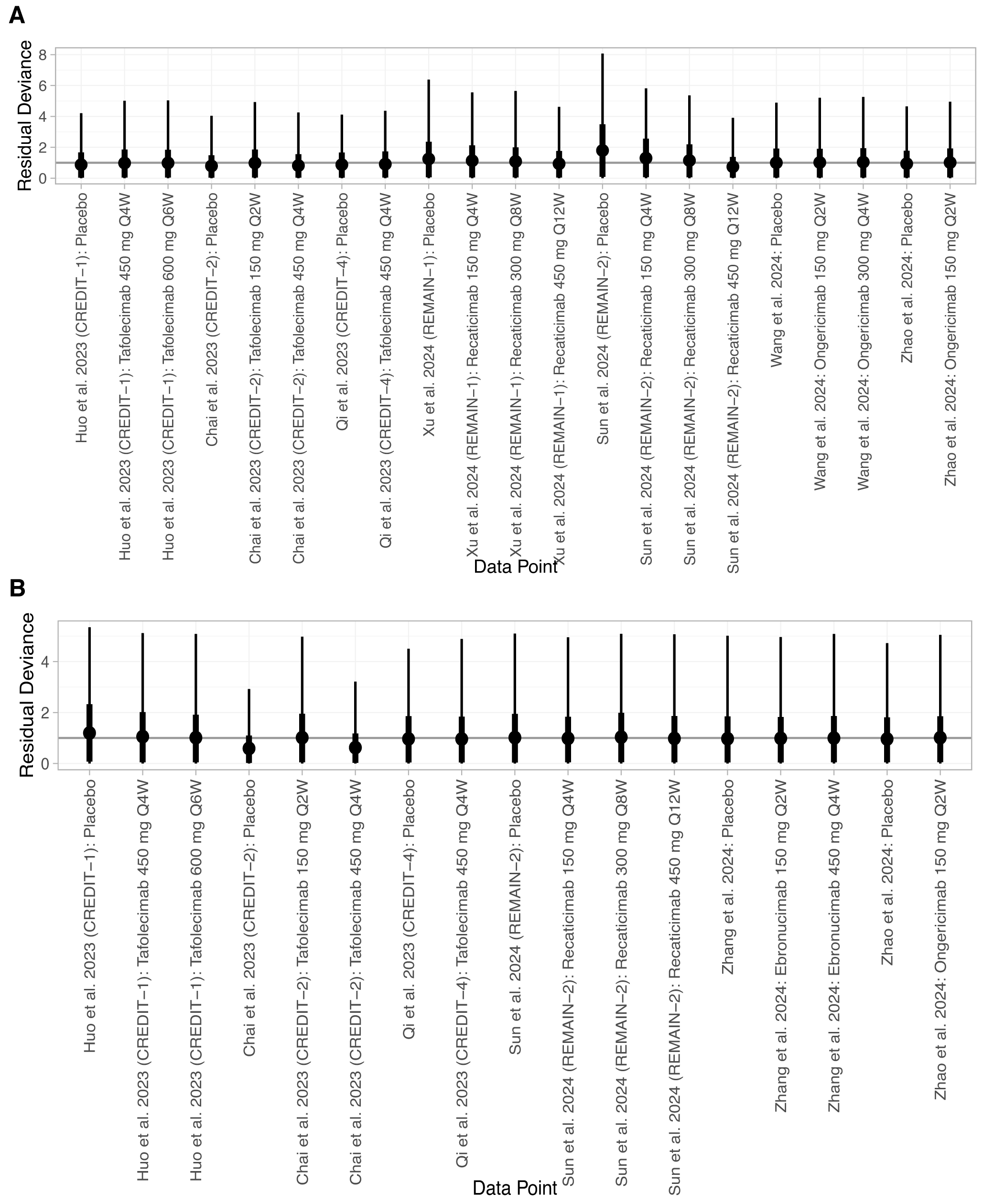


# Figure S7. Residual deviance contributions of (A) LDL-C outcome in random-effects model; (B) TEAEs outcome in fixed-effects model. *LDL-C in fixed-effects model: residual deviance = 28.8, pD = 15, DIC = 43.8; in random-effects model: residual deviance = 21.4, pD = 18.7, DIC = 40.1. TEAEs in fixed-effects model: residual deviance = 16.7, pD = 15.3, DIC = 32; in random-effects model: residual deviance = 17, pD = 16.5, DIC = 33.5.*

**Figure S8.** Meta-regression analysis of clinical efficacy in LDL-C outcome. **(A)** other forms of hyperlipidemias; **(B)** follow-up duration, weeks; **(C)** baseline LDL-C level, mmol/L. MH, mixed hyperlipidemia; PH, primary hypercholesterolemia; Q2, 4, 6, 8, 12W, every 2, 4, 6, 8, 12 weeks.

**Figure S9.** Meta-regression analysis of clinical safety in TEAEs outcome. **(A)** other forms of hyperlipidemias; **(B)** follow-up duration, weeks; **(C)** baseline LDL-C levels, mmol/L. MH, mixed hyperlipidemia; PH, primary hypercholesterolemia; Q2, 4, 6, 8, 12W, every 2, 4, 6, 8, 12 weeks.


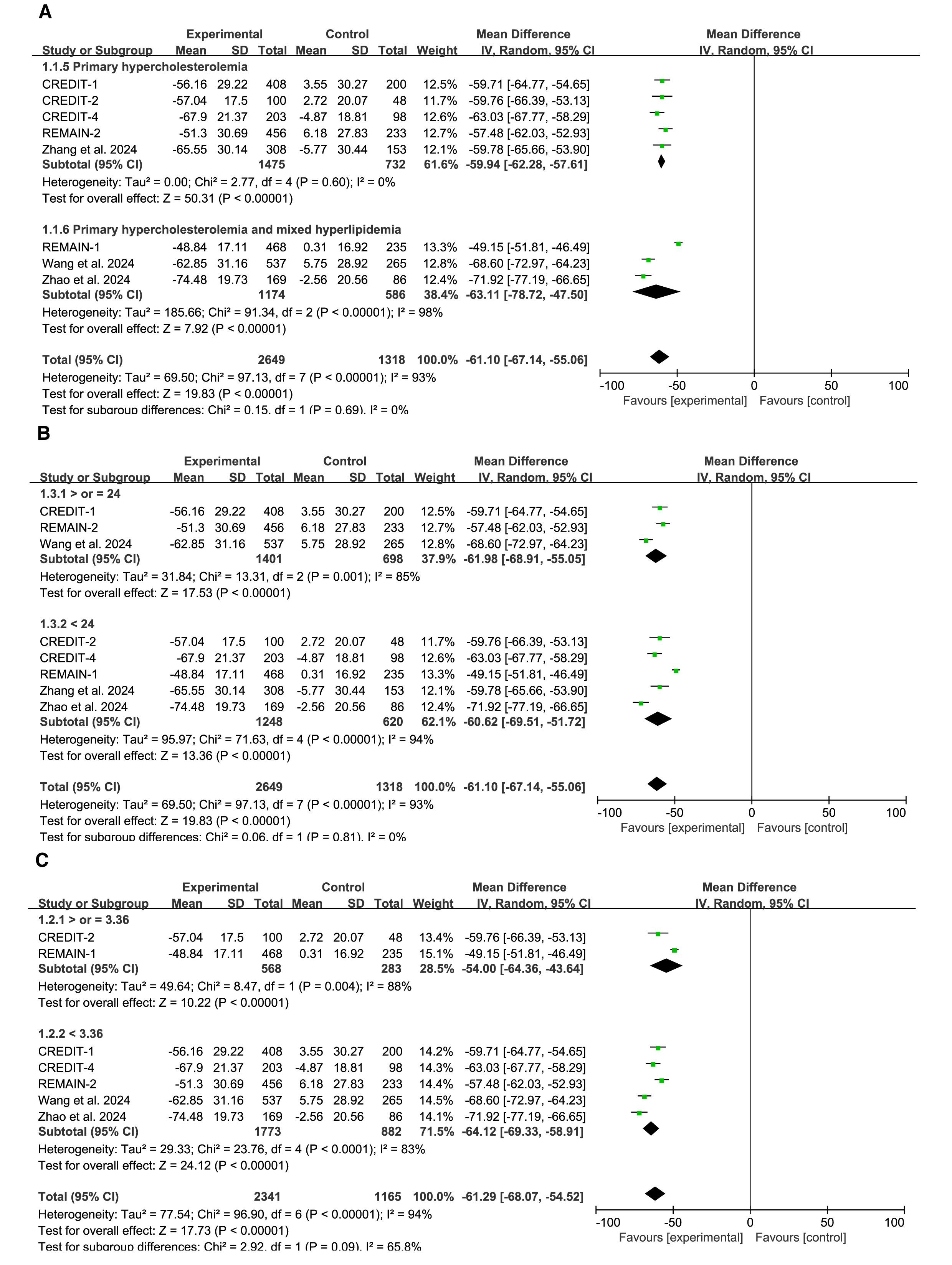


**Figure S10.** Subgroup analysis of PCSK9 inhibitors versus placebo in LDL-C outcome, %. **(A)** other forms of hyperlipidemias; **(B)** follow-up duration, weeks; **(C)** baseline LDL-C levels, mmol/L.


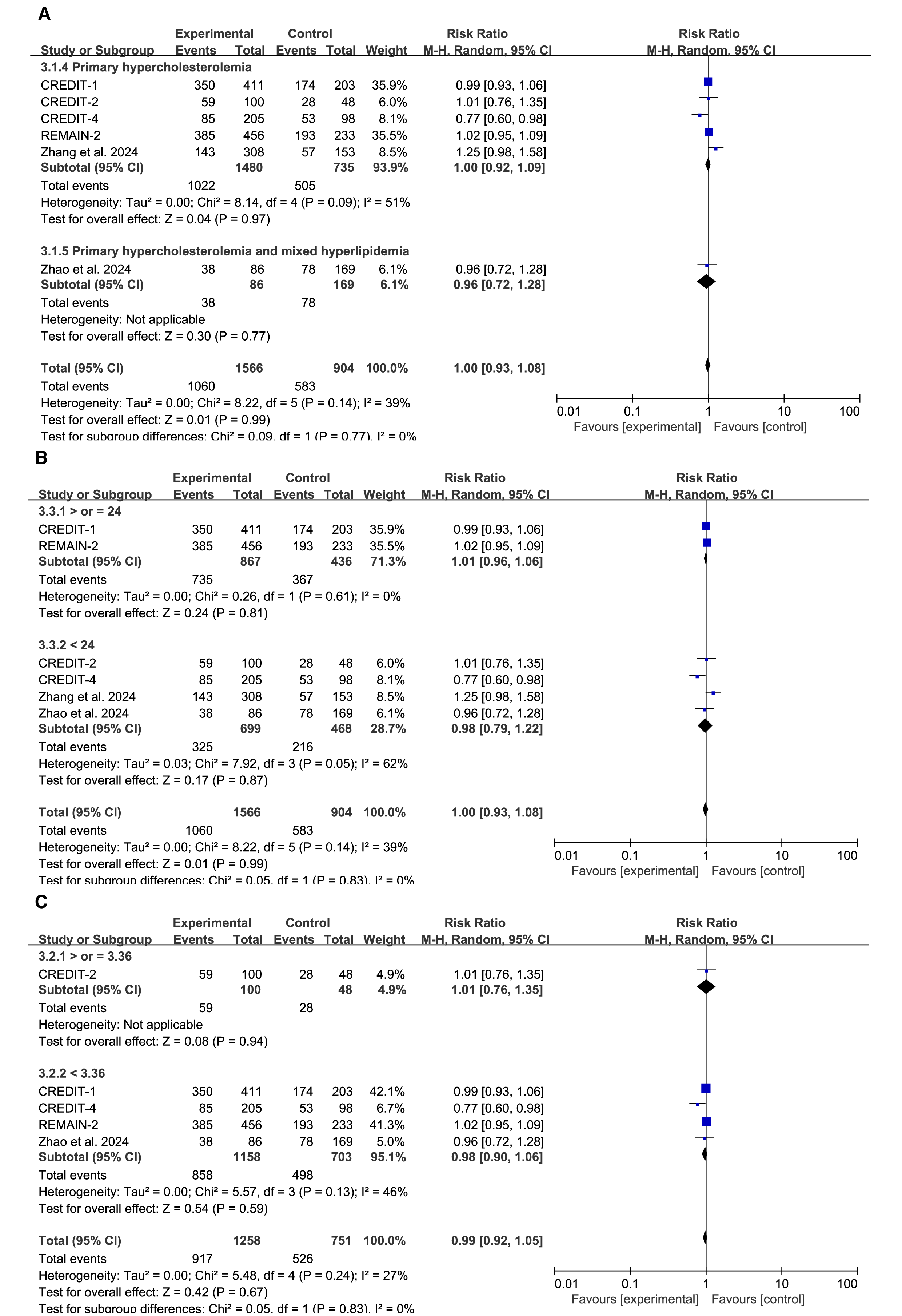


**Figure S11.** Subgroup analysis of PCSK9 inhibitors versus placebo in TEAEs outcome. **(A)** other forms of hyperlipidemias; **(B)** follow-up duration, weeks; **(C)** baseline LDL-C levels, mmol/L.

**Figure S12.** Sensitivity analysis of clinical efficacy by excluding high-risk studies. **(A)** LDL-C, %; **(B)** ApoB, %; **(C)** Lp(a), %. CI, confidence interval; MD, mean difference; Q2, 4, 6, 8, 12W, every 2, 4, 6, 8, 12 weeks.


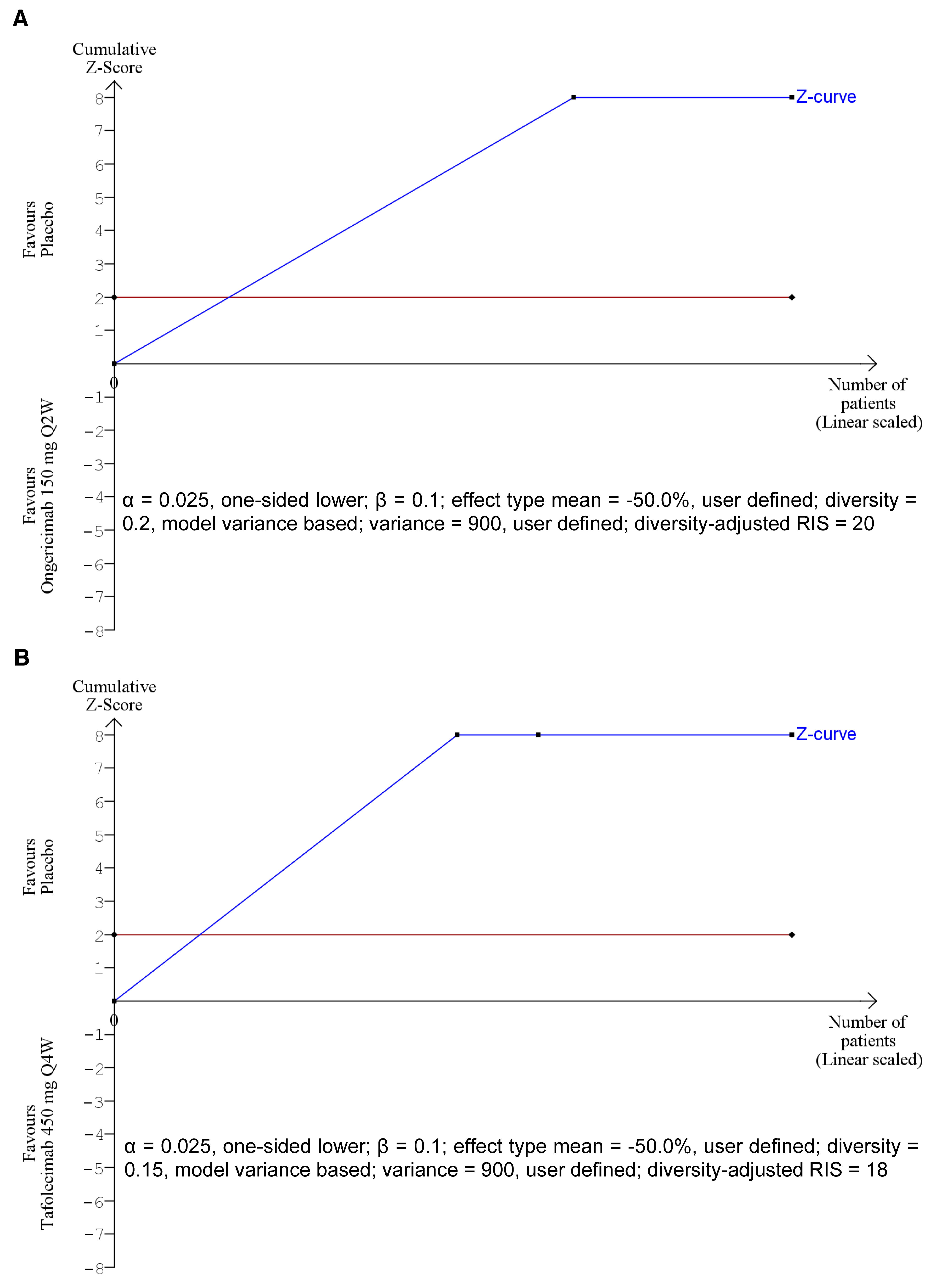


**Figure S13.** Trial sequential analysis of LDL-C percentage reduction. **(A)** Ongericimab 150 mg Q2W versus Placebo; **(B)** Tafolecimab 450 mg Q4W versus Placebo. The blue solid line represents the cumulative Z curve, while the red solid line denotes the threshold line at Z = 1.96. The mean effect differences and variances were estimated conservatively based on the RCT evidence – *1. Sabatine MS, Giugliano RP, Keech AC, et al. Evolocumab and Clinical Outcomes in Patients with Cardiovascular Disease. N Engl J Med. 2017 May 4;376(18):1713-1722.; 2. Robinson JG, Farnier M, Krempf M, et al. Efficacy and safety of alirocumab in reducing lipids and cardiovascular events. N Engl J Med. 2015 Apr 16;372(16):1489-99.*


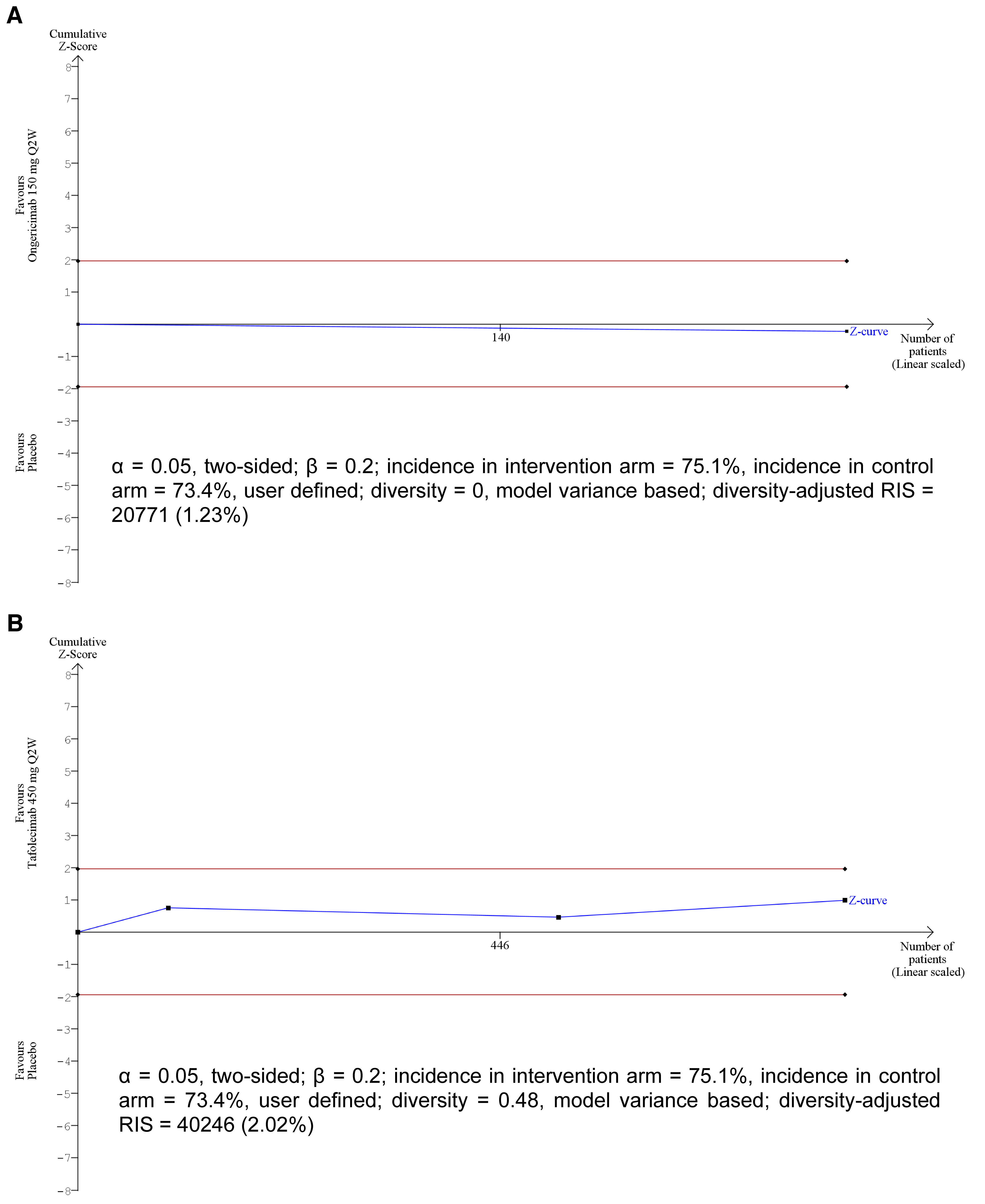


**Figure S14.** Trial sequential analysis of TEAEs incidence. **(A)** Ongericimab 150 mg Q2W versus Placebo; **(B)** Tafolecimab 450 mg Q4W versus Placebo. The blue solid line represents the cumulative Z curve, while the red solid line denotes the threshold line at Z = 1.96. The incidence was based on the meta-analysis evidence - *Choi HD, Kim JH. An Updated Meta-Analysis for Safety Evaluation of Alirocumab and Evolocumab as PCSK9 Inhibitors. Cardiovasc Ther. 2023 Jan 4;2023:7362551. doi: 10.1155/2023/7362551.*
